# Developing the Protocol Infrastructure for DNA Sequencing Natural History Collections

**DOI:** 10.3897/BDJ.11.e102317

**Published:** 2023-10-27

**Authors:** Giada Ferrari, Lore Esselens, Michelle L Hart, Steven Janssens, Catherine Kidner, Maurizio Mascarello, Joshua V Peñalba, Flávia Pezzini, Thomas von Rintelen, Gontran Sonet, Carl Vangestel, Massimiliano Virgilio, Peter M Hollingsworth

**Affiliations:** 1 Royal Botanic Garden Edinburgh, Edinburgh, United Kingdom Royal Botanic Garden Edinburgh Edinburgh United Kingdom; 2 Royal Museum for Central Africa, Tervuren, Belgium Royal Museum for Central Africa Tervuren Belgium; 3 Royal Belgian Institute of Natural Sciences, Brussels, Belgium Royal Belgian Institute of Natural Sciences Brussels Belgium; 4 Meise Botanic Garden, Meise, Belgium Meise Botanic Garden Meise Belgium; 5 Leuven Plant Institute, Department of Biology, Leuven, Belgium Leuven Plant Institute, Department of Biology Leuven Belgium; 6 Museum für Naturkunde, Leibniz Institute for Evolution and Biodiversity Science, Berlin, Germany Museum für Naturkunde, Leibniz Institute for Evolution and Biodiversity Science Berlin Germany; 7 Royal Museum for Central Africa, Department of African Zoology, Tervuren, Belgium Royal Museum for Central Africa, Department of African Zoology Tervuren Belgium

**Keywords:** Museomics, hDNA, biodiversity genomics, natural history collection sequencing

## Abstract

Intentionally preserved biological material in natural history collections represents a vast repository of biodiversity. Advances in laboratory and sequencing technologies have made these specimens increasingly accessible for genomic analyses, offering a window into the genetic past of species and often permitting access to information that can no longer be sampled in the wild. Due to their age, preparation and storage conditions, DNA retrieved from museum and herbarium specimens is often poor in yield, heavily fragmented and biochemically modified. This not only poses methodological challenges in recovering nucleotide sequences, but also makes such investigations susceptible to environmental and laboratory contamination. In this paper, we review the practical challenges associated with making the recovery of DNA sequence data from museum collections more routine. We first review key operational principles and issues to address, to guide the decision-making process and dialogue between researchers and curators about when and how to sample museum specimens for genomic analyses. We then outline the range of steps that can be taken to reduce the likelihood of contamination including laboratory set-ups, workflows and working practices. We finish by presenting a series of case studies, each focusing on protocol practicalities for the application of different mainstream methodologies to museum specimens including: (i) shotgun sequencing of insect mitogenomes, (ii) whole genome sequencing of insects, (iii) genome skimming to recover plant plastid genomes from herbarium specimens, (iv) target capture of multi-locus nuclear sequences from herbarium specimens, (v) RAD-sequencing of bird specimens and (vi) shotgun sequencing of ancient bovid bone samples.

## Introduction

### Natural history collections as a resource for genomic science

There are more than one billion specimens representing ca. two million species stored in natural history collections worldwide (Wheeler et al. 2012, Yeates et al. 2016, Johnson et al. 2023). These collections span a wide geographical and temporal range and represent a globally distributed biorepository. They house biological specimens representing the world’s known species, along with many specimens representing undescribed species awaiting taxonomic recognition and formal taxonomic descriptions (Bebber et al. 2010). First and foremost, these natural history collections were established to support understanding of species diversity and distributions (Miller et al. 2020) and the vast majority of specimens housed in these repositories were collected to preserve their appearance and morphological features; most specimens were not collected with DNA sequencing in mind (Roycroft et al. 2022).

Until recently, the recovery of DNA sequences from museum specimens was challenging and prone to very high rates of failure or requiring laborious protocols for successful recovery of minimal quantities of nucleotide sequence data (Lalueza-Fox 2022). However, with the development of improved sequencing technologies and protocols, there is now a rapid surge of interest in the field of museomics (Card et al. 2021, Raxworthy and Smith 2021). Considerable attention is being given to unlocking DNA data at a large scale, capitalising on the centuries of effort that have gone into the acquisition of biological specimens for natural history collections (Hebert et al. 2013, Folk et al. 2021). At a very practical level, natural history collections provide access to easy-to-retrieve and often well-identified specimens. This contrasts with the considerable challenges and costs associated with obtaining freshly-collected material for DNA analyses, such as field collecting costs, the cost of preparing voucher specimens and the difficulties of accessing taxonomic expertise to ensure accurate biosample identifications (Hebert et al. 2013, Camacho et al. 2018). These challenges are exacerbated for taxa occurring in remote and/or poorly-studied locations (Wandeler et al. 2007) or areas that are difficult to access because of political instability or conflict (Burrell et al. 2015). Furthermore, where taxa or populations have been lost in the wild, natural history collections are often the only genetic resource for extinct and endangered species (Wandeler et al. 2007, Clewing et al. 2022). Beyond these practical benefits of sampling museum specimens for DNA, there are also the unique scientific opportunities that come from being able to undertake time-series analyses capitalising on the temporal component of natural history collections; DNA sequencing of these collections can provide direct windows into evolutionary processes and patterns of adaptation and evolutionary change (Holmes et al. 2016) and the trajectory of species of conservation concern (Nakahama 2021, Jensen et al. 2022). Likewise, sequencing specimens from natural history collections can also provide insights into the dynamics of associated organisms, such as pathogens, parasites and other intimately-connected species residing in or on museum specimens (Bieker et al. 2020, Ferrari et al. 2020, Ristaino 2020, Raxworthy and Smith 2021, Speer et al. 2022).

### Storage and preservation of museum specimens

The global collection of preserved natural history specimens contains a diverse set of samples encompassing a multitude of different tissue types and preservation methods (Carter and Walker 1999). Major collections that are stored dry include pressed plant and fungal herbarium specimens, pinned insects, bones, teeth, shells, skins and hides. Specimens that are stored dry are often subjected to direct heat treatment during the drying process and, in some cases, chemical treatments. For instance, plant material from the Tropics was often immersed in alcohol prior to direct-heat drying to prevent specimens rotting in humid environments (Hodge 1947). Animal skins may have been prepared using a wide range of techniques, including air drying, salting, tanning and chemical treatments, such as arsenic (McDonough et al. 2018). Likewise, a substantial proportion of natural history museum specimens are stored wet, in spirit-based fixatives, including whole specimens, individual organs and other body parts of a diverse array of animals; many of these wet museum collections (especially fish, reptiles and amphibians) were often fixed with, or may still be preserved in, formalin (Hahn et al. 2021).

### Properties of DNA in natural history collections and implications for sequencing museum specimens

The DNA within natural history museum specimens has distinct properties from the DNA in freshly-collected material which has practical implications for recovering sequence data. From a biochemical perspective, DNA isolated from natural history collection specimens shares many similarities with ancient DNA (aDNA). Characteristically, aDNA is highly fragmented and biochemically damaged, often present in small quantities and subject to contamination from the environment and human handling. In the absence of the enzymatic repair mechanisms of living cells, DNA is subject to hydrolysis, oxidation and cross-linking (Dabney et al. 2013a), processes that can be accelerated by high temperatures, extreme environmental pH, humidity and the presence of microorganisms (Willerslev and Cooper 2005, Willerslev et al. 2007). Hydrolysis and oxidation can lead to depurination, which results in DNA strand breakage (Lindahl 1993). As a consequence, aDNA is typically no longer than 150 bp (Green et al. 2009).

These degradation processes for aDNA also occur to greater or lesser degrees in museum specimens. Various studies of DNA degradation in natural history collections have shown that DNA fragmentation can occur rapidly after death (Sawyer et al. 2012), with a wide range of reported fragment lengths, including frequent reports of fragment lengths < 100 bp (McDonough et al. 2018, Canales et al. 2022, Mullin et al. 2023). There is an imperfect relationship between specimen age and levels of fragmentation, with some studies showing a correlation between specimen age and fragment length (McCormack et al. 2016, Weiß et al. 2016, Mullin et al. 2023), whereas others did not (Sawyer et al. 2012). The factors affecting the rate of fragmentation are complex (Kistler et al. 2017) and include differences between genomes (e.g. mtDNA sequences showing slower degradation than nuclear sequences (Heintzman et al. 2014)), differences between tissue types (Kistler et al. 2017, Andreeva et al. 2022) and differences due to the environmental conditions at the site of specimen preparation and different storage environments and preservation methods (Brewer et al. 2019, Card et al. 2021, Mullin et al. 2023). For instance, museum storage conditions may impact on biomolecule degradation, with temperature and humidity influencing levels of preservation (Kistler et al. 2017, Brewer et al. 2019). Likewise different preservation methods themselves impact on the preservation and recoverability of nucleotide sequences: several preparation techniques involve heat, which accelerates DNA hydrolysis resulting in fragmentation (Lindahl 1993, Willerslev and Cooper 2005). Formalin fixation is a commonly-used technique for wet-mounted specimens and, especially if unbuffered, can cause a number of reactions, including DNA fragmentation via acid-driven hydrolysis and DNA-protein cross-linking that results in PCR inhibition (Brutlag et al. 1969, Gilbert et al. 2007b). Treatment of bones with ammonium solutions and various tanning agents has also been reported to reduce DNA yields from museum specimens (Vuissoz et al. 2007, Tarbet Hust and Snow 2021). Finally, pest-control treatments of collection specimens can also impact the recovery of DNA (Espeland et al. 2010, Töpfer et al. 2011).

Following the fragmentation of DNA in museum specimens, there is a consequential and associated loss of DNA as DNA fragments diffuse away from specimens. Kistler et al. (2017) proposed a model by which DNA fragmentation occurs rapidly after death before slowing down, then bulk diffusion leads to the decay of DNA concentration through time. This highlights the importance of tissue types which create closed systems that minimise DNA loss for the retention and recovery of nucleotide sequences (e.g. dense bone tissue, seed tissue).

There are several consequences of this fragmentation and loss of DNA. The first is that experimental effort may be expended which ultimately leads to a failure to recover DNA sequence data due to low endogenous DNA content. The second is that DNA sequence data may be recovered, but be misleading due to contamination. The potential for contamination in sequence data from museum specimens is substantial. The low concentrations of fragmented endogenous DNA in museum specimens represent an initial low signal-to-noise ratio and a high potential for contamination from a wide variety of sources, including:

1. Biological material on the specimen (surface contaminants and biological materials associated with specimen preparation);
2. High concentrations of DNA from fresh samples and their amplification products processed in the same facility which represent an important source of contamination when handling degraded DNA. Such contaminant DNA may be present in higher concentrations than the DNA in historic samples and this is exacerbated by subsequent PCR being biased towards higher-quality DNA;
3. General contamination in the processing lab, including sources of contaminating DNA from specimen handling, laboratory reagents and aerosols in the wider environment.

At best, contamination reduces sequencing efficiency for endogenous DNA and requires greater sequencing efforts at higher costs. What is more problematic is the generation of erroneous data where misleading biological inferences are made from undetected contamination (Yeates et al. 2016).

A more generic source of error, but one to which museum-derived sequences are particularly susceptible, is problems stemming from low coverage of sequence reads due to low DNA concentration. This can result in misleading inference; for example, failure to recover both alleles in diploid heterozygotes leading to an overestimation of homozygosity at some loci and in some specimens (Ewart et al. 2019) or, more generally, the introduction of noise due to miscalls which may simply override any weakly-resolved genuine signal in the data.

Above and beyond the challenges of recovering reliable sequence data from low concentrations of fragmented endogenous DNA is the possibility of post-mortem modifications leading to artefactual substitutions in the recovered DNA sequences. Post-mortem hydrolytic deamination causes base modifications, primarily affecting cytosine (Orlando et al. 2021). Uracil, the deamination product of cytosine, causes the misincorporation of adenine during DNA amplification. This results in C to T substitutions in the deaminated strand and G to A substitutions in the complementary strand of DNA (Briggs et al. 2007, Brotherton et al. 2007). This deamination of cytosine resulting in C to T and G to A substitutions during amplification can, if unchecked, lead to a systematic misleading signal in the data. Sequences from different biological samples may share nucleotide changes due to these miscoding lesions which may be misinterpreted as genuine biological similarities. The accumulation of miscoding lesions at the end of DNA molecules is a feature of aDNA (and used as an important parameter for the validation of aDNA data authenticity (Briggs et al. 2007, Green et al. 2009)); however, they do tend to be less common in studies focusing on natural history collections. DNA deamination correlates with specimen age and there is an expectation for more recently collected museum specimens to show limited impacts of deamination-related substitutions, compared to centuries-old specimens which, in turn, are expected to show substantially lower impacts than those found in ancient DNA material (Weiß et al. 2016, Kistler et al. 2017, Canales et al. 2022).

### Outstanding challenges to the routine sequencing of museum specimens

There has been a recent shift in the field of museomics from small-scale studies, often with high rates of failure, to increased success rates and a growth of increasingly-ambitious studies aiming to liberate sequence data at a large scale from museum collections (Hebert et al. 2013, Alsos et al. 2020, Kates et al. 2021, Mullin et al. 2023). The complexity of museum collections themselves and the variation in specimen ages, tissue types, preservation methods and storage conditions, preclude simple universal high-throughput methods. Nevertheless, there is considerable scope for continued optimisation of approaches and community development and dialogue around appropriate and optimal working standards. Within this general challenge, specific areas for development in making the recovery of sequence data from museum specimens more reliable and routine include:

- Deciding when it is appropriate to sample museum specimens: Development of guiding principles to facilitate sampling decisions that support specimen utilisation, but avoid unnecessary and unproductive destructive sampling;
- Minimising the risks of contamination and production of erroneous sequence data: Guidance and utilisation of appropriate laboratory infrastructure and data verification steps;
- Maximising the recovery of endogenous DNA sequences: Optimisation of protocols to improve the efficiency and efficacy of different widely used techniques.

These three topics are addressed in subsequent sections of this paper.

## Deciding when it is appropriate to sample museum specimens for DNA sequencing

Destructive sampling poses a dilemma between damaging a specimen for research utilising existing protocols and preserving the specimen for future and improved methodologies (McDonough et al. 2018). Thus, museomic studies need to consider the likelihood of successfully obtaining DNA sequences, the scientific insight that can be obtained, the amount of material needed with regards to specimen and collection size and the potential benefits of postponing sampling to await future methodological advances. Where available, low(er) value specimens in museum collections represent useful material for protocol development and testing prior to destructive sampling on material of higher value. Many museum collections contain samples of limited taxonomic value (e.g. large volumes of sterile material), samples with poor meta-data or specimens which have abundant duplicate material. Such specimens represent more suitable candidates for experimentation than high-value, important and/or unique individual specimens, such as type material.

To minimise damage to specimens, a number of minimally or non-destructive sampling protocols for collection material have been proposed. DNA extraction protocols for ancient and historic DNA have been optimised to obtain good DNA yields from small amounts of material, for example, as low as two milligrams of dry plant tissue (Latorre et al. 2020) or one insect leg (e.g. Cavill et al. (2022)). In terms of less invasive sampling, approaches include sampling the embedding fixative solution of wet collection specimens (Rayo et al. 2022), rubbing an eraser over herbarium specimens (Shepherd 2017) and gentle digestion followed by drying of teeth (Rohland et al. 2004), whole insect specimens (Gilbert et al. 2007a, Korlević et al. 2021) or herbarium material (Sugita et al. 2020). Such approaches can be extremely useful for many applications, although ‘non-destructive’ sampling often yields lower DNA amounts, limiting genetic analyses to low coverage of DNA sequences or high copy-number regions, such as organellar genes.

Maximising the use of museum specimens, including for genomic analyses, while minimising unnecessary destruction of precious samples thus reflects a balancing act. This is made particularly difficult, as often the greatest scientific returns will come from the specimens that are most valuable. For instance, type specimens will almost always have significant constraints on their use, which may act as a barrier to inclusion in genomic studies. On the other hand, effective minimally destructive sequencing of type specimens provides a direct connection between genomic data and the application of a species name and, hence, represents a significant scientific benefit, particularly for taxonomic and systematic studies. There is a general point, that while sampling a museum specimen for genomic analysis usually results in something being taken away from the specimen to obtain DNA, it can also result in something extremely useful being added, in terms of critically important genomic data which may add considerable value to the specimen (i.e. the concept of the extended specimen; Webster (2017)).

Guidance on best practice standards and processes for the access and transfer of samples for genomic analysis is given by the Consortium of European Taxonomic Facilities (2015) and de Mestier et al. (2022). However, from a perspective of the impacts on the specimens themselves, it is not surprising, given the rapidly-evolving state of the field and the complexity of choices regarding different collections and different approaches, that there are no community standards to guide *when* it is appropriate to destructively sample specimens. There are various policy documents to guide decision-making at institutional levels and several useful more general perspectives (e.g. Freedman et al. (2018), Austin et al. (2019), Pálsdóttir et al. (2019)). To further facilitate the navigation of ‘when and how’ to sample, we outline ten key principles which can usefully be followed by researchers and assessed by curators in guiding when to undertake destructive sampling of specimens for genomic analyses:

1. Assess the scientific merit of the planned genomic project; ensure there is a clear likely benefit prior to commencing destructive sampling and that the resulting data will be informative and of sufficient resolution to tackle the question at hand;
2. Always adopt a minimally destructive approach for genomic studies of museum specimens unless there is a clear surplus of available tissue, such as extensive duplicate specimens or ‘sacrificial’ specimens available for experimentation;
3. Utilise alternative options to destructively sampling important specimens if available (e.g. make use of any reliable previously-sampled tissues or previous DNA extracts, adopt a non-destructive sampling approach, if applicable);
4. If multiple tissue types are available, consider the likely success rates for the different tissues and weigh this against their respective morphological impacts on the specimen in choosing which tissue to sample;
5. Seek to maximise the reusability of the data from destructive sampling: consider methodologies which give maximum amounts of data which will be of use for multiple downstream applications;
6. Seek to maximise the reusability of DNA from destructive sampling: adopt methods of handling and storing DNA extracts to maximise their preservation and reuse potential to minimise further need for specimen sampling;
7. Process samples following appropriate laboratory controls and with clear data-verification steps to ensure that the resulting data have maximum reliability and value;
8. Evaluate the feasibility of success prior to destructive sampling of valuable specimens in terms of protocol efficacy, researcher capability and laboratory suitability and only proceed where the likely chances of success and resulting scientific benefits outweigh the costs of any destructive sampling;
9. Report successes and failures to guide future optimisation of protocols and decision-making regarding destructive sampling;
10. Ensure appropriate accessibility of the resulting sequence data and linkages and connections between the data and the specimens they were derived from, to ensure that specimen sampling for genomic analyses results in added value to the specimen itself.

## Minimising the risks of contamination and generation of erroneous sequence data

### Historic DNA versus ancient DNA versus modern optimally-preserved DNA

The opportunities arising from the sequencing of museum specimens have attracted researchers from different backgrounds and fields. On the one hand, sequencing the degraded DNA in museum specimens has long been a focus for aDNA researchers. Ancient DNA techniques involve working with low concentrations of highly-degraded DNA in specialist laboratories with strict guidelines and meticulous anti-contamination precautions (Gilbert et al. 2005, Llamas et al. 2017). These techniques and working practices developed for aDNA allow the recovery of genetic material up to and over one million years old (van der Valk et al. 2021, Kjær et al. 2022). On the other hand, taxonomists, systematists and other researchers focusing on contemporary biological samples often process larger numbers of samples in more general laboratory facilities working with tissue samples preserved in a fashion aimed at maintaining high concentrations of non-degraded DNA.

The DNA in the majority of natural history museum specimens sits at the interface of aDNA and non-degraded DNA samples and is classed as historic DNA (hDNA), more formally defined as DNA from specimens archived in museum collections that were not originally intended as genetic resources (Billerman and Walsh 2019, Raxworthy and Smith 2021, Irestedt et al. 2022). This classification recognises that museum specimens, typically collected over the last 250 years, have different properties to specimens recently collected and preserved for DNA analyses and ancient specimens deposited in nature over millennia (Raxworthy and Smith 2021, Wandeler et al. 2007). This distinction between ancient and historic DNA is useful, although it should be noted that the line between archaeological specimens, natural history collections and even biobank material is a blurred one. As noted previously, several factors other than age influence DNA preservation, such as temperature, substrate, taphonomic conditions and specimen preparation and storage. Thus, a permafrost-preserved archaeological sample may be a better DNA source than a heat-dried or chemically-treated museum voucher. Because of the lack of *a priori* information regarding the magnitude of DNA degradation in historical collection material, a pragmatic working assumption for hDNA material is to assume damage and fragmentation, as well as environmental contamination (Latorre et al. 2020).

### Current operational practices for processing hDNA

The maximally effective recovery of hDNA is dependent on determining the appropriate levels of stringency of laboratory practices, which minimise risks of contamination, while at the same time, being sufficiently scalable to allow maximum utilisation of the vast resources of specimens available in museum collections. Thus, while utilising dedicated aDNA facilities (Fulton 2012) and the full suite of operational precautions for processing aDNA material is the conservative option, it will not be appropriate in all cases (Raxworthy and Smith 2021) and full aDNA facilities and protocols for all specimens would represent a substantial constraint on sample processing.

During the preparation of this paper, discussions amongst the authors and an informal survey of colleagues working in a range of organisations involved in sequencing museum specimens, revealed a wide range of operational practices. These ranged from processing samples in the same laboratories as fresh tissue, through to dedicated hDNA (or low-copy) laboratories, through to only ever using fully-equipped aDNA facilities for processing museum specimens. A multitude of factors were articulated as underlying the decision-making of which facilities to use for processing hDNA samples, including:

- Resource constraints (money and/or space) precluding establishment of a dedicated facility;
- Desire to use existing facilities in standard labs to enable processing of large numbers of samples;
- Controls in place for data authentication and/or stringent cleanliness conditions in standard labs considered adequate to negate the need for a dedicated facility for hDNA samples;
- Individual preferences of researchers determining where samples are processed without clear institutional policies;
- When both aDNA and hDNA samples have to be processed in the same institution, the aDNA laboratory being used exclusively for aDNA samples, with museum specimens processed elsewhere due to concerns that the higher concentrations of DNA from museum specimens may lead to contamination problems in the aDNA lab.

### Laboratory set-ups and workflows for hDNA sequencing

A general observation noted by several researchers familiar with working with non-degraded DNA samples was a lack of clarity over what an optimal laboratory set-up would look like for hDNA analysis. To facilitate evaluation of options for contamination control and the practicalities of laboratory set-ups, we outline below the headline infrastructure and working practices of an aDNA facility and a basic hDNA facility. We also list contamination-limiting recommendations for processing degraded material in existing, more general, laboratories.

**Ancient DNA facility**

The most critical component of setting up an aDNA laboratory (Gilbert et al. 2005, Fulton 2012, Knapp et al. 2012, Llamas et al. 2017) is a strict separation of pre- and post-PCR areas. This includes no movement of equipment, reagents, consumables or samples from post- to pre-PCR. Similarly, scientists should not move from the post- to pre-PCR without showering and changing clothes. The dedicated aDNA pre-PCR facilities are physically isolated from any post-PCR area and are used for sample processing, DNA isolation and setting up of sequencing library and PCR reactions. These reactions are moved to post-PCR facilities at the first DNA amplification step and post-amplification products can be handled normally in shared laboratory facilities. Amplification products, fresh biological material and modern DNA samples should never be introduced into the aDNA facilities. Additionally, the dedicated aDNA laboratory should be fitted with a positive pressure, HEPA-filtered air system and UV lights for daily sterilisation of all surfaces. Equipment, tools and working surfaces should be cleaned daily or after every use with a 1-2% sodium hypochlorite solution or a surface decontaminant such as DNA Away™ or DNA Exitus™. Plastic consumables should be UV-sterilised and only filter tips should be used. Everything that is introduced into the laboratory should be decontaminated. The aDNA laboratory should be accessed through an antechamber where incoming reagents and consumables are sterilised (and ideally introduced through a dedicated UV-hatch) and PPE donned (overalls with hood, hairnets, facemasks, face shields, double gloves and shoe covers or dedicated shoes). Destructive sampling and sample powdering should take place in a separate room inside a PCR cabinet or dead-air box. Samples and DNA extracts should be handled exclusively in HEPA-filtered laminar flow cabinets, equipped with UV lamps (and should be cleaned and sterilised after every use). A separate cabinet for DNA-free applications (aliquoting reagents, preparing master mixes) is needed. Additional good working practices include processing samples in small batches and the inclusion of several non-template negative controls for DNA isolation and library preparation, as well as dividing reagents into smaller aliquots.

**Historic or low-copy DNA facility**

Natural history collection material can be handled in aDNA laboratories. However, aDNA laboratories and their upkeep can be prohibitive in cost; therefore, institutions working exclusively on natural history collections may choose a less stringent set-up for a dedicated hDNA pre-PCR facility. This would usually be located in existing rooms rather than a purpose-built laboratory. Not requiring a positive pressure air system and a laboratory antechamber allows for more flexibility in the choice of location (e.g. repurposing of laboratory spaces) and significantly reduces the costs. It should be noted that if an hDNA facility is being established by repurposing an existing laboratory, thorough cleaning with sodium hypochlorite of all surfaces is essential and new, dedicated equipment should be bought. Similarly to aDNA facilities, all work should take place inside UV-fitted PCR cabinets, and destructive sampling and sample powdering should take place in a separate room or at least in a separate cabinet. Additional UV lamps for surface decontamination may be fitted; however, repeated UV exposure is damaging to laboratory equipment, increasing upkeep costs. Cleaning routines and good practices as described for aDNA facilities should be implemented or adapted as best as possible, most importantly the separation of pre- and post-PCR working areas. In Suppl. material 1, we outline a relatively inexpensive (ca. €86K) and pragmatic equipment list for establishing a dedicated low-copy facility for hDNA processing, that is flexible enough to be installed without extensive building works.

**Contamination-limiting measures for working in existing facilities**

Due to space or financial constraints or because of the need for higher throughput, it remains the case that many institutions may decide against an aDNA or hDNA facility and process natural history collection material in existing laboratories alongside fresh biological material. Fresh material and especially its amplification products, represents a common source of contamination for historic material. Thus, the separation of pre- and post-PCR areas remains essential, although the routes to achieving this can be varied. At the very least, thermocyclers should always be located in the post-PCR area and movement of samples, reagents, consumables and equipment between pre- and post-PCR should be avoided or limited as best as possible. Additionally, pre-PCR work on collection material should be carried out in dedicated laminar flow hoods (to be cleaned and UV-sterilised regularly) with dedicated tools and reagents.

### Data verification and contamination controls

In addition to the physical layout of laboratories, data verification and contamination controls include:

*Verification checks against reference libraries*: The likelihood of being misled by contamination in the sequencing of museum specimens is a function of the stringency of the controls and the complexity of the detection task. Data-authentication steps can be relatively straightforward, where there is an *a priori* expectation of the sequence to be recovered and an existing reference resource to check it against. Thus, studies like large-scale DNA barcoding projects, are intrinsically well-suited to contamination checks, with small regions of DNA being recovered from individual museum specimens which are usually identified to species level and whose identity can be checked by sequence cluster placement in existing DNA barcode reference libraries such as BOLD (Ratnasingham and Hebert 2007, Ratnasingham and Hebert 2013) if there is sufficient coverage of the study taxon. Likewise, genome skimming studies or target capture-based recovery of organelle genomes can benefit from systematic comparisons of extracted barcode loci against barcode reference libraries (Timmermans et al. 2016, Alsos et al. 2020), as well as wider checks against the growing reference datasets of organelle genomes (e.g. Li et al. (2021)). The data verification checks become more challenging where the density of reliable reference data for comparison is lower and the complexity of the sequence data produced is higher. There are various major infrastructure projects underway driving the development of reference libraries which will facilitate these checks. The International Barcode of Life Project (iBOL) continues to accelerate the production of standardised DNA barcode data. BIOSCAN, the current phase of the iBOL project, aims to generate barcode coverage for two million species by 2027 (Hobern 2021) and the associated development of national and regional barcode initiatives (such as BIOSCAN Europe https://www.bioscaneurope.org/) have a strong focus on the production of tightly-curated reference barcode libraries. Another complementary large-scale biodiversity genomics infrastructure project, the Earth Biogenome Project, has the goal of producing reference genomes for all eukaryotic species (Lewin et al. 2022) and various large-scale geographically-focused projects are underway (The Darwin Tree of Life Project Consortium 2022); the increased density of high-quality reference genomes will greatly support data verification and assembly of the short-read sequences from museum specimens as is already possible with human DNA (de Filippo et al. 2018).

*Ordering samples to avoid closely-related taxa being in adjacent wells*: The most difficult contamination to spot is from closely-related samples, as even detailed analysis and comparisons with reference samples may not flag contaminants. Where there is a mixture of closely- and more distantly-related taxa being processed, a simple option is to order samples to maximise the likelihood of adjacent well contamination being detectable, by minimising the presence of closely-related specimens in adjacent wells.

*Negativecontrols*: The inclusion of non-template negative controls at the DNA isolation and library preparation/PCR step is essential for ensuring that the pre-PCR facilities and reagents are sufficiently clean. Negativecontrols should also be taken through all post-amplification steps, sequenced and included in the data analysis.

*Sequencing strategies*: Jumping PCR (Kircher et al. 2012) and index switching during cluster generation on the Illumina sequencing platform (van der Valk et al. 2020) can result in reads potentially being assigned to the wrong sample. This normally does not pose a problem for libraries generated from high-quality DNA, but can introduce an artefactual contamination into degraded DNA libraries. This can be overcome by unique dual-indexing of libraries, a common practice in aDNA experiments. Using unique indices in both library adapters allows the detection and removal of these chimeric PCR products. Unique dual-indexing, if not repeated within the same laboratory, also allows monitoring of potential cross-contamination between projects.

*DNA repair*: To mitigate against misleading inference due to post-mortem DNA modifications, enzymatic treatment of DNA extracts can be undertaken, for instance with the USER reagent (New England Biolabs), a mix of uracil–DNA–glycosylase (UDG) and endonuclease VIII (Orlando et al. 2021). On the other hand, if post-mortem DNA modifications are of importance for authentication (e.g. for aDNA studies), then avoidance of this step is equally important.

*Data authentication and validation*: In addition to checks against reference libraries, various bioinformatic pipelines and workflows support the verification and authentication of degraded DNA sequences. These include estimating contamination by screening for unexpected results such as 'heterozygosity' of haploid genomes (mitogenomes, plastomes) (Krause et al. 2010, Renaud et al. 2019). These approaches, based on deviations of expected ploidy, require sufficiently high sequencing coverage, but do not necessitate any *a priori* knowledge of the origin of the contamination (Peyrégne and Prüfer 2020). Likewise, testing for the presence of sequencing artefacts due to post-mortem DNA damage (C to T and G to A misincorporations) at the read ends can be undertaken using specific software such as mapDamage2 (Jónsson et al. 2013) and PMDtools (Skoglund et al. 2014). This is particularly relevant to older samples.

## Maximising the recovery of endogenous DNA sequences

Over the last decade, there has been a constantly expanding set of literature outlining new developments which contribute to making the recovery of sequence data from museum specimens more cost-effective and routine (Knyshov et al. 2019). These include breakthroughs in sample and tissue types that were previously considered intractable, including significant improvements in prospects for recovery of genomic data from formalin-fixed tissues (Ruiz-Gartzia et al. 2022). For instance, Straube et al. (2021) used aDNA extraction protocols and single-stranded DNA library preparation to achieve high rates of success in recovering endogenous DNA from wet museum collections of a range of vertebrate taxa including formalin-fixed samples, followed by a target-capture approach to recover almost complete mitogenomes from a subset of these samples. Likewise, Hahn et al. (2021) used a hot-alkaline lysis approach for DNA extraction, followed by whole genome sequencing to successfully recover mitochondrial genomes and up to 3X nuclear genome coverage from a diverse range of formalin-preserved vertebrate tissue specimens and outlined a framework to guide decision-making for genomic studies utilising spirit-preserved collections.

The generation of guidelines and decision-making frameworks to support more routine recovery of sequence data from collections is of considerable value as the field of museum collection sequencing expands (McDonough et al. 2018). Of particular use are very large-scale studies processing thousands of specimens which offer the potential for general predictions to emerge regarding the likelihood of recovery of useful sequence data. For instance, Kates et al. (2021) processed nearly 8000 herbarium specimens from a range of angiosperm families from six different herbaria in the United States and evaluated factors influencing DNA recovery and sequencing success using a target-capture approach. They showed the strongest predictor of success was related to taxonomic group (some families performed better than others, likely due to physical or chemical properties of the samples). There was a more limited correlation between DNA yield or sequencing success on the one hand, and the age of specimens or their greenness on the other ('greenness' is an indicator of the preservation process as green specimens are less likely to have been subject to heat or ethanol treatment during drying). Likewise, extensive studies processing thousands of preserved insect specimens from museum collections for DNA barcoding have developed efficient workflows that recover cytochrome oxidase 1 (CO1) sequences for many specimens using primer cocktail sets and Sanger sequencing for younger specimens, coupled with more intensive multiplex PCR and high-throughput sequencing platforms to recover barcode sequences from older specimens (Prosser et al. 2016, Levesque-Beaudin et al. 2023, Santos et al. 2023).

Table 1 highlights a selection of studies outlining recent progress, breakthroughs and protocol developments which support the more routine recovery of genomic data from museum specimens.

A general challenge for the effective recovery of endogenous DNA from museum specimens is the frequent low complexity of libraries caused by PCR and cleaning steps modifying the relative abundances of the original DNA fragments during library preparation (Casbon et al. 2011). This leads to the formation of artefactual PCR duplicates that may bias sequencing results, decrease final coverage and increase sequencing costs (Rochette et al. 2023). PCR duplicates can be removed during bioinformatic analysis (Marx 2017); however, given the low concentration and quality of DNA from museum specimens, it may be valuable to enhance the library complexity prior to sequencing. To this end, amounts of starting material can be increased where possible (Fu et al. 2018) and single-tube library preparation methods can be used (e.g. Carøe et al. (2018), Kapp et al. (2021)). PCR conditions can also be adapted, for example, by selecting polymerases that are suited to copy degraded DNA templates with good fidelity and without a severe tendency to preferentially amplify DNA templates that are shorter or with higher GC content (Aird et al. 2011, Dabney and Meyer 2012, Seguin-Orlando et al. 2015). Protocols for archival specimens generally perform amplifications in several independent PCR reactions with minimal numbers of cycles (van der Valk et al. 2021, Irestedt et al. 2022) and with sequencing efforts proportional to library complexity (Daley and Smith 2014). Finally, sequencing by synthesis with 50 to 150 cycles format and single-end mode is usually more cost-effective with short degraded fragments (Raxworthy and Smith 2021); however, choosing more cycles or a paired-end mode may be valuable if fragment length distributions are used to filter out contaminants.

## Case study overview

To provide further details on protocol development for particular genomic approaches and their success in application to different taxonomic groups, we present a series of case studies undertaken at different institutions as part of the EU SYNTHESYS+ project. Each focuses on protocol practicalities for the application of different mainstream methodologies to museum specimens including: (i) shotgun sequencing of insect mitogenomes (Museum für Naturkunde Berlin), (ii) whole genome sequencing of insects (Royal Museum for Central Africa), (iiii) genome skimming to recover plant plastid genomes from herbarium specimens (Meise Botanic Garden), (iv) target capture of multi-locus nuclear sequences from plants (Royal Botanic Garden Edinburgh), (v) RAD-sequencing of bird specimens (Royal Belgium Institute for Natural Sciences) and (vi) shotgun sequencing of ancient bovid bone samples (Royal Belgium Institute for Natural Sciences).

## Case study 1: Assessing the potential of rapid shotgun sequencing for the recovery of mtDNA genomes from pinned insect specimens

### Introduction

The world’s entomological collections hold more than half a billion pinned (dried) insect specimens (Short et al. 2018), representing an enormous genetic and genomic resource, for tackling a wide range of questions of great scientific and societal importance, not least of which is better understanding global insect declines (Card et al. 2021). For most species-rich insect taxa with a fundamental role in terrestrial ecosystems (pollinators, food sources, parasitoids, primary consumers etc.), reliable diversity estimates for megadiverse tropical regions are at best available for just a few subgroups. By mobilising sequence information (especially from types) from natural history collections, it will become much easier to identify new species as well as synonyms, and thus speed up the process of biodiversity discovery, as well as building a database for DNA-based species biomonitoring.

This potential has received increasing attention in recent years (Raxworthy and Smith 2021) and several studies have looked at the recovery of DNA with a specific focus on DNA isolation protocol optimisation to increase recovery of sequence data and minimise damage to specimens for NGS sequencing (e.g. Patzold et al. (2020), Korlević et al. (2021)). In many instances, even a limited amount of mtDNA data, such as short DNA barcodes, will be sufficient for resolving taxonomic issues and characterising patterns of species diversity in highly-diverse insect taxa (Yeo et al. 2020). Here, we evaluate the performance of shotgun sequencing (low-coverage genome sequencing) of museum specimens with a wide age range from three key insect taxa to explore the utility of a fast, generic and inexpensive approach to obtain mtDNA data from natural history collections to support DNA-based biodiversity inventories and biomonitoring.

### Materials and Methods

Specimens (n = 70 in total) were selected from a genus (or two closely-related genera) from each of three major holometabolan insect groups (Diptera, Hymenoptera, Lepidoptera) with a collecting date ranging from 1891 to 2015 (132-8 years in collection storage). The taxa (Diptera: *Sarcophaga*; Lepidoptera: *Eudonia*, *Scoparia*; Hymenoptera: *Xylocopa*) are actively studied by the respective curators at Museum für Naturkunde and the availability of at least one reference mitogenome was an additional core criterion. One leg each was used for DNA isolation using the Qiagen Investigator Kit. Multiplexed libraries for paired-end Illumina sequencing were prepared for each sample with the “NEXTflex Rapid DNA Seq Kit 2.0” and “NEXTflex HT Barcodes” (Bioscientific/PerkinElmer). DNA input varied between 1 and 300 ng (10 ng or less for > 75% of samples). As the DNA of all samples was expected to be strongly degraded, the protocol was adapted accordingly (no shearing and bead-size selection, adjustment of bead buffer - sample ratio for clean-up after adapter ligation). Library quality, size and quantity were determined with a TapeStation (D1000 Kit Kit/ Agilent) and Qubit 2.0 Fluorometer (dsDNA HS Assay Kit/Thermo Fisher Scientific). Libraries were pooled, based on these parameters and low-coverage test sequenced on an Illumina Miseq (PE150). Based on the results from the test sequencing, 12 libraries were subjected to a booster PCR to increase quantity. Libraries were re-pooled, based on library parameters (concentration) and test sequencing results (number of reads) and the resulting pool was sequenced on an Illumina Nextseq (PE150). The cleaned and de-multiplexed reads were mapped against mtDNA genomes of the respective target taxon using the MITObim pipeline (Hahn et al. 2013).

### Results and Discussion

**Figure 1.**
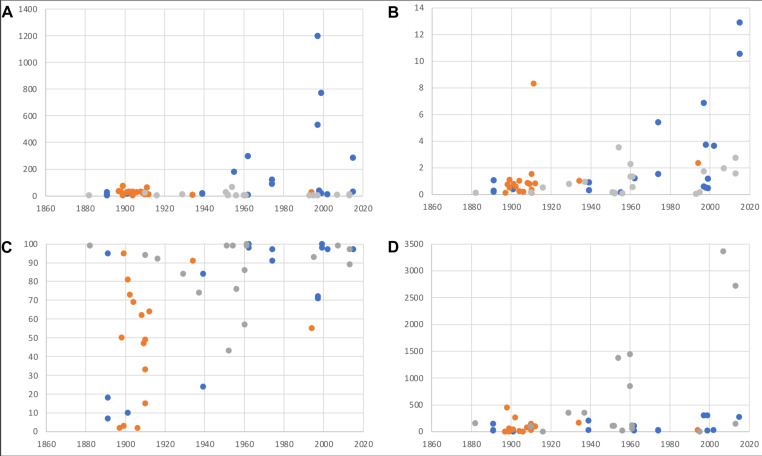
Scatter plots of DNA and sequence recovery from pinned insect specimens by age and taxon (blue - *Eudonia*/*Scoparia*, grey - *Sarcophaga*, orange - *Xylocopa*). Specimen age (in years) is on the x axis in all panels. **A** Total DNA yield (ng). **B** Number of sequencing reads. **C** Completeness of the mitogenomes (%). **D** Coverage (n).

The amount of recovered DNA ranged from 3 ng to 1.2 µg (Fig. 1a). The highest amount of DNA for samples collected before 1950 was 75.6 ng and all samples yielding more than 100 ng were collected after 1950, but note that 50% of these younger samples did not exceed 100 ng either. The overall highest amounts of DNA were recovered from samples collected between 1990 and 2000. This pattern was also recovered when comparing read number and sample age (Fig. 1b) and apart from one exception, all samples with > 2 mio. reads were collected after 1970 and > 10 mio. reads were only exceeded in the two youngest samples (2015). Mapping reads against mitogenome sequences of the target taxa obtained from GenBank recovered a minimum of 14,652 bp for *Xylocopa* (Hymenoptera), 15,299 bp for *Eudonia* (Lepidoptera) and 15,667 bp for *Sarcophaga* (Diptera). The published mitogenome of *Xylocopa* is partial with only 14,655 bp, which might explain the shorter assemblies for that genus. Completeness and coverage varied strongly amongst samples. All samples with a completeness below 50% (7-33%, n = 8; Fig. 1c) were collected before 1950, while no sample collected after 1950 had less than 70% completeness. The pattern for coverage was different, as both a relatively low coverage of < 50 and a high coverage > 100 were found throughout the entire age range of samples (Fig. 1d). An extremely high coverage of > 1000 was only found in *Sarcophaga*.

**Success rate in relation to sample age**

DNA degradation is affected by several variables, such as initial preservation of samples and storage conditions, which in themselves are highly diverse in many aspects (temperature, humidity, pest control chemicals). As the effect of these factors accumulates over time, an obvious assumption is that DNA degradation will be worse in older specimens and sequence recovery consequently more difficult, as has been shown in a recent systematic study using NGS methods (Mullin et al. 2023). Our results support this generalised conclusion to some degree: the oldest samples (collected before 1950) yielded consistently low amounts of DNA and only one of the old specimens yielded more than 2 mio. sequence reads. However, the mixed patterns for (relatively) younger samples support the notion that the initial preservation of samples is also contributing to the degradation of DNA, as about 50% of specimens collected after 1950 yielded about the same magnitude of DNA and sequencing reads as the older samples, which is notable given that some of the poorly-performing younger samples were collected as recently as the year 2000. A rapid degradation of DNA following death was also found by Sawyer et al. (2012) and Kistler et al. (2017). The completeness and coverage of recovered mitogenomes showed a relationship with sample age, albeit with high levels of variation. Thus, although all samples with low completeness were older than 80 years, there was substantial variation in the completeness of mitogenomes amongst the oldest samples (collected < 1920).

Overall, no sample failed entirely as measured by obtaining reads from mtDNA. One sample did not yield any reads at all after the booster PCR, but the same sample worked (albeit with only ca. 81,000 reads) for the library prepared without additional PCR amplification.

In contrast to age, variation in success rate does not seem to differ amongst taxa, if variation amongst collecting dates is taken into account. Almost all samples of *Xylocopa*, for example, were collected before 1940 and about 75% in 1912 or before, and the apparently lower success rate for *Xylocopa* could be explained by age alone. Similarly, the species and consequently specimens used in this study differed in body size, which was not controlled for here and, as the total amount of DNA recovered is expected to be dependent on tissue input, that value cannot be directly compared meaningfully between specimens of different taxonomic groups.

**Effectiveness in terms of costs and time of shotgun sequencing**

Shotgun sequencing is a straightforward and technically undemanding approach by NGS standards. In recent years, it has also become ever more cost effective as measured by cost per bp. For example, in this study, each sample yielded on average 1.6 mio. reads and sequencing costs per sample were ca. €32 (price as of December 2022). These costs could be further reduced using different sequencing platforms (e.g. Illumina Novaseq).

Interestingly, to date, a limited number of studies have used shotgun sequencing for museomics in insects and only one has targeted mtDNA in particular for taxonomy in a specimen-based approach. All types of Australian prionine longhorn beetles were shotgun sequenced by Jin et al. (2020), leading to a major revision of their taxonomy. The methodological study by Timmermans et al. (2016) aimed to show the applicability of shotgun sequencing in museum specimens, but by a metagenomic approach that combined DNA extracts without individual sample indexing. Cong et al. (2021) used a shotgun approach for taxonomic purposes in North American butterflies on a large number of specimens, but targeted mainly nuclear genes and their study relied on creating a reference genome from a modern sample first. Other shotgun sequencing studies have aimed to place enigmatic taxa on the tree of life (Twort et al. 2021) or explore population genetic structure (Cridland et al. 2018) or species conservation (Mikheyev et al. 2017) by focussing on deeper sequencing of few (2-29) museum specimens. While these studies focused on aspects of species biology, Mullin et al. (2023) focussed on > 100 specimens of one species of bumble bee from the UK to study DNA preservation in museum specimens. Their results also show that there is great potential in the use of museum specimens of pinned insects for biodiversity genomics research.

A frequently-used alternative to shotgun sequencing for recovering genomic data from museum specimens is target capture (e.g. Blaimer et al. (2016), Mayer et al. (2021)). It has mostly been used for generating substantial amounts of nuclear data, but as yet not so much for mitogenomics (but see Knyshov et al. (2019)). An advantage of target capture is the significant increase in sequencing efficiency, as target genes will make up a much larger proportion of reads, allowing the pooling of more samples and consequently bringing down sequencing costs per sample. The downside of this approach is that it only works reliably up to a genetic distance of ca. 12% between target DNA and bait sequences. Custom bait sets need to be created at a rather low taxonomic level (generally genus or even species groups, depending on genetic divergence between species). This is a more acute problem for mtDNA capture, as genetic divergence in mtDNA is about four times higher than in nuclear DNA. Baits can either be ordered custom made, which is expensive (ca. €120/reaction as per manufacturer’s instructions or between €5-15 if 8-24 libraries are captured with the amount of baits delivered for one reaction) or PCR generated (Knyshov et al. 2019). While the latter approach is more cost-effective, with costs of €25-39 in total (including sequencing per sample according to Knyshov et al. (2019)), it does add to the workload.

For both low-coverage shotgun sequencing and target capture, there is a trade-off between costs, time and sequencing success (measured by the completeness of the target sequence(s)), which is likely to tilt towards bait capture when the aim is to sequence a large number of closely-related samples. However, if the aim is to target a larger range of taxa at the genus level and beyond for taxonomic purposes, such as DNA barcoding, then shotgun sequencing has the edge in our opinion due to its relative ease and the universality of approach. As sequencing costs are still on a downward trajectory, the cost balance is likely to be tilted further in its favour in the future.

*Main findings and recommendations*:

- A shotgun approach is particularly appropriate for obtaining (mtDNA) data for a wide range of different taxa with relatively little effort in the lab, which makes it highly useful for taxonomy and providing reference sequences from type material. If the aim is to generate complete datasets from many individuals of closely-related species, bait capture might be a viable alternative;
- Older samples will often require more sequencing effort to obtain the same amount of data as more recent specimens. If the main aim is the generation of DNA barcodes for taxonomic purposes, this should not be overly relevant in practice;
- When using a shotgun approach, using a leg is sufficient to obtain an adequate amount of data for taxonomic purposes at least from medium-sized to large (> 10 mm) specimens, which should make it easier for curators to give permission for destructive sampling;
- Crucially, when adding to collections, sample preservation should be optimised in the field in order to avoid heavy DNA degradation before specimens become museum specimens.

*Data availability*: The raw sequencing data from this case study has been deposited at the European Nucleotide Archive (ENA) under project PRJEB59182 with accession IDs ERS14475133 - ERS14475206.

## Case study 2: Genomic vouchering in insect museum collections: the quest for a pragmatic approach to routine, large-scale genotyping

### Introduction

The costs directly related to genomic library preparation and sequencing represent one of the main limiting factors hampering the whole genome sequencing (WGS) of large numbers of museum specimens. Until recently, the partial sequencing of genomes, via approaches, such as reduced representation libraries (Ewart et al. 2019) or mitochondrial genomics (Timmermans et al. 2016), was considered as the only suitable approach to build up relatively large genomic datasets. However, the rapid technological advances of the past few years have led to a substantial reduction in costs, so that the routine WGS of vouchers represents a new, exciting perspective for the valorisation of museum collections (e.g. Crampton-Platt et al. (2016), Malakasi et al. (2019), Strijk et al. (2020)). In this case study, we present a feasibility study on the routine collection of genomic data from insect museum vouchers. Here, with "routine genotyping" we refer to standard and commonly-used wet-lab pipelines for WGS rather than to the more elaborate, expensive and technically-challenging protocols which are used to recover highly-degraded DNA (reviewed in Orlando et al. (2021)).

### Materials and Methods

**Comparative performances of commercially-available DNA extraction kits**

The performance of commercial DNA extraction kits (see below) was compared in a pilot study targeting the Royal Museum of Central Africa (RMCA) collections of “true” fruit flies (Tephritidae, Diptera) and African hoverflies (Syrphidae, Diptera). We selected three to six specimens from seven sample groups dating from 2008 to 2016. These included three Tephritidae (*Zeugodacuscucurbitae* Coquillett, *Bactroceradorsalis* Hendel and *Dacusbivittatus* Bigot) and two Syrphidae (*Eumerus* sp. and *Ischiodonaegyptius* Wiedemann) species; all specimens were stored in 100% ethanol at -20°C, except *Ischiodonaegyptius* which was pinned and preserved at room temperature. Digestions in lysis buffers were implemented on whole bodies for all specimens. For comparative purposes, we also processed forelegs of a separate set of specimens. The lysates obtained from each specimen were divided into four aliquots and the DNA purified using spin columns from the DNA extraction kits listed in Table 2 following the manufacturers' instructions. The experimental design was based on 30 whole specimens and 18 legs (two negative controls were also included); these samples were processed through 200 spin columns from four different extraction kits. The concentration of each DNA extract was measured using a Qubit 3 fluorometer (HS DNA Kit, Thermo Fisher Scientific) and the total amount of DNA was inferred from the final elution volume, which in all cases was 100 µl.

**Relationships between voucher DNA quality and WGS performance**

**Figure 2.**
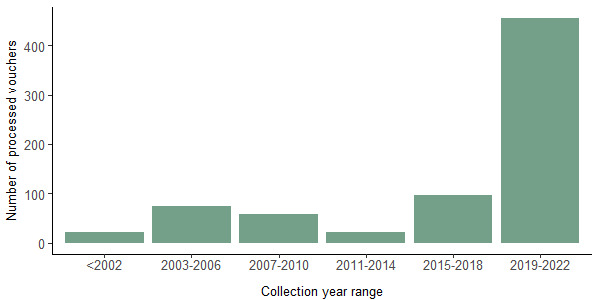
Age distribution of processed specimens of fruit flies and hoverflies.

To assess the relationship between WGS performance and (a) voucher age and preservation and (b) DNA quality and quantity, we targeted a total of 732 insect vouchers archived in the collections of RMCA collected from 1997 to 2022 (Fig. 2) and preserved either in ethanol at -20°C (n = 651), pinned at room temperature (n = 14) or dried DNA stored at room temperature (n = 67). All DNA extractions were performed using the DNeasy Blood and Tissue Kit (Qiagen catalogue nr. 0148945380). We quantified the amount of DNA extracted as measured by a Qubit 4 fluorometer (HS DNA Kit, Thermo Fisher Scientific) and the quality of DNA via DNA fragment size distributions as measured using the DNF-930 dsDNA Reagent Kit (75 bp – 20000 bp) on the fragment analyser of the Genomics Core (Leuven, Belgium). These vouchers were considered as suboptimal for genomic analyses as preliminary pilot tests (results not shown) revealed generally lower concentrations of DNA and higher levels of DNA fragmentation compared to freshly-processed specimens.

Based on DNA concentrations (above or below 7.0 ng/µl) and DNA fragmentation (> 350 bp or highly fragmented defined as < 350 bp), samples were submitted to Berry Genomics (n = 563) for standard library preparation or to Novogene (n = 81) for low input DNA library preparation, respectively. All samples were sequenced at 10x coverage on an Illumina NovaSeq platform (150 PE reads, 6 Gb raw data output/sample). Quality parameters of extracted DNA and WGS data of specimens originating from five insect genera and more than 70 different species were collected (see Table 3 and Suppl. material 2).

### Results and Discussion

**Figure 3.**
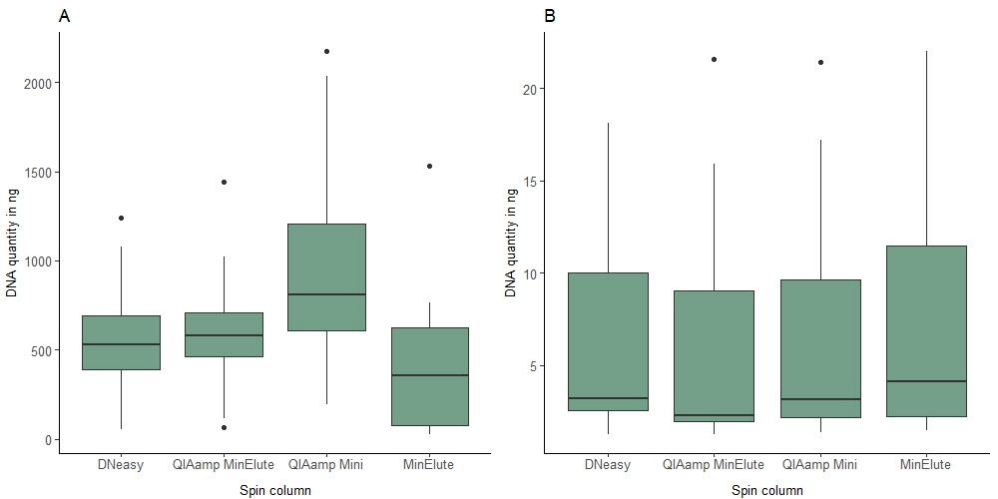
Boxplots of DNA yields from replicated elutions of Tephritidae and Syrphidae of **A** whole body digestions and **B** leg digestions per DNA extraction method (Qiagen, DNeasy Blood and Tissue Kit, cat. 69506; QIAamp DNA Mini, cat. 51304; QIAamp DNA Micro, cat. 56304; MinElute PCR Purification, cat. 28004).

Overall, our DNA extractions provided detectable DNA in 720 specimens out of 732 (98.4%) while WGS data could be obtained for 644 museum vouchers (88.0%). The different DNA extraction methods gave broadly similar yields, albeit with a somewhat lower recovery of DNA from whole-body extractions using the MinElute kit. Overall, there was a heterogeneous recovery of DNA yields across specimens (Fig. 3), with values ranging from 57.8 to 153.0 ng for whole bodies. As expected, due to the lower amount of tissue, DNA amounts recovered from legs was comparably lower and ranged from 1.3 to 22.0 ng. Based on minimising costs, we adopted the kit with the lowest price (DNeasy Blood and Tissue Kit) for routine processing of vouchers from the target insect collections.

**Figure 4.**
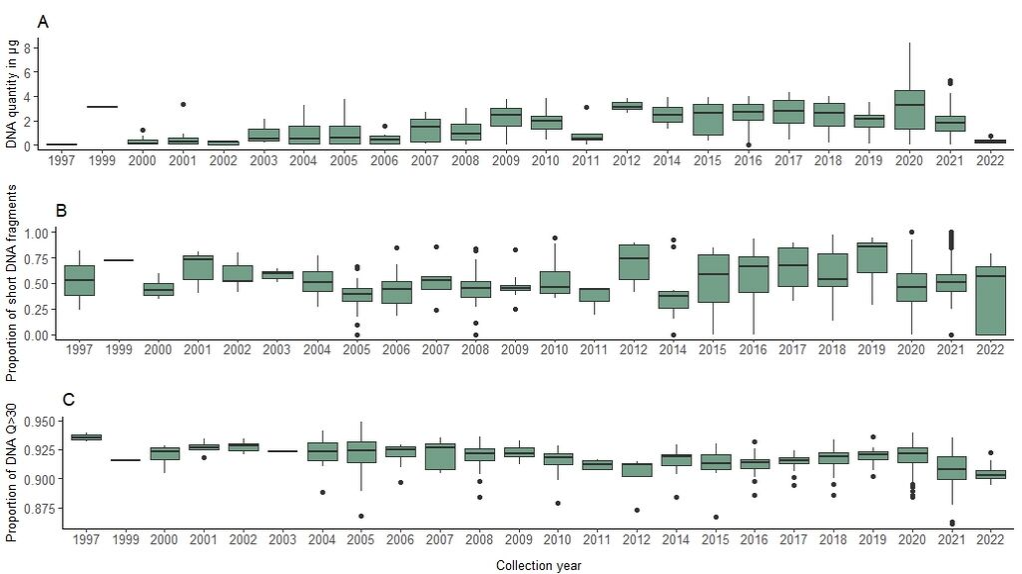
Boxplots per collection year for Tephritidae and Syrphidae specimens extracted with the DNeasy Blood and Tissue Kit (Qiagen): **A** DNA quantities (calculated from concentration measured with Qubit 4.0); **B** the proportion of DNA fragments between 35 and 350 bp (measured with Fragment Analyzer (DNF-930 dsDNA Reagent kit)); **C** proportion of sequenced reads with Q > 30.

Our results show a general trend of decreasing recovery of DNA from older specimens compared to younger specimens (Fig. 4a). In contrast, our assessment of DNA quality as estimated by fragment lengths of the DNA extracts and Phred score (Q > 30) of raw sequences lacks any clear temporal trend, with degraded DNA with short fragment lengths and low quality reads recovered across the range of specimen ages (Fig. 4b and c).

Sub-optimal or low-quality DNA from museum specimens is often not directly suitable for genetic/genomic analyses (Besnard et al. 2016). However, our results suggest that standard DNA extraction, based on commercially available kits followed by WGS at 10x genome coverage, represents a cost/time-effective, pragmatic approach to the routine, large-scale genotyping of insect vouchers collected over the past two decades. The majority of samples processed in this analysis were of material stored in ethanol. The samples that were pinned at room temperature (n = 14) or stored as dried DNA at room temperature (n = 67) showed similar DNA quantity and quality results as the DNA from specimens stored in ethanol.

The DNA of these diverse and heterogeneously collected samples, even if generally suboptimal in terms of concentration, fragmentation and contamination, still allowed recovery of substantial amounts of quality reads (Q > 30) of potential use for genomic research. This general approach needs to be complemented with more specialised and time/cost-demanding procedures for highly-degraded DNA from older specimens. A two-step approach, including the use of commercial kits and methods outlined here allows for rapid screening of younger specimens and reserving the more intensive protocols (also including aDNA methodologies) for older specimens represents a pragmatic, cost-effective route to the routine genotyping of our insect collections.

*Main findings and recommendations*:

- The DNeasy Blood and Tissue Kit (Qiagen) provided a cost-effective method of extracting DNA from museum specimens aged one to 25 years;
- These recently-collected samples, although containing fragmented DNA, represent a tractable tissue source for large-scale sequencing projects;
- For older material, the use of low input library preparation for highly-fragmented and low concentration DNA extracts is recommended.

*Data availability*: The data and meta-data from this case study are documented in Suppl. material 2.

## Case study 3: Genome skimming as a tool to recover whole plastid genomes from threatened Central African timber species

### Introduction

**Figure 5.**
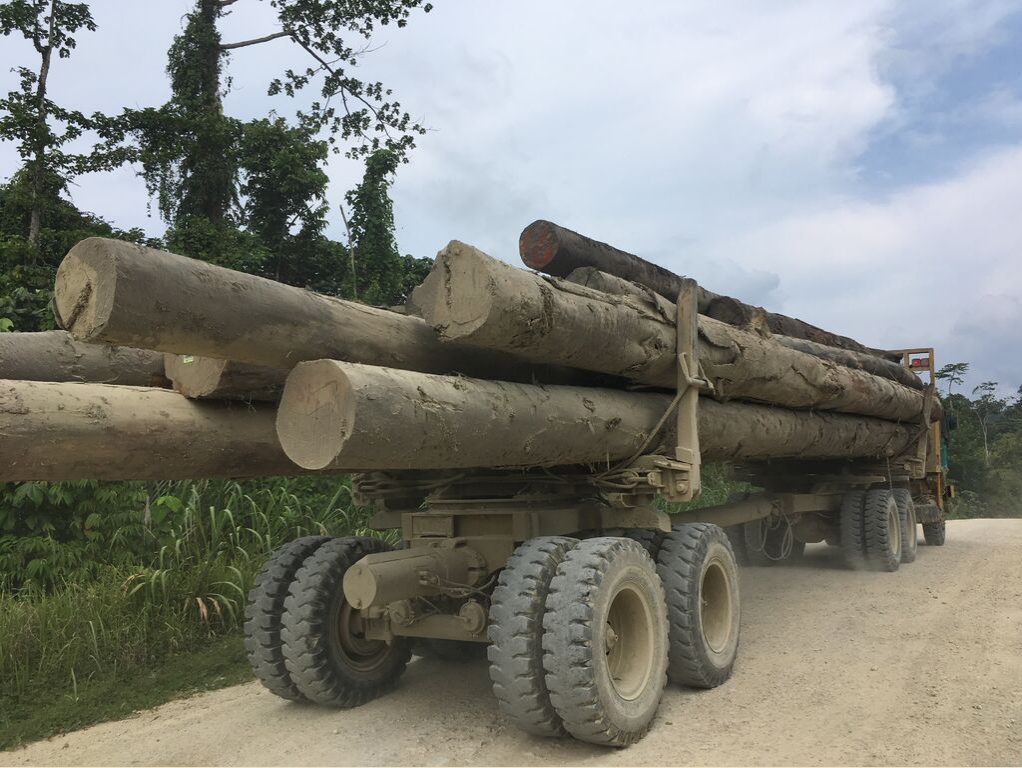
Transportation of large tree trunks from the forest to enter the international timber trade.

Worldwide, multiple tree species used for timber production are under severe threat (Fig. 5). Despite a restriction on logging concessions and the improvement of forestry laws, recent studies show that, for example, in the Democratic Republic of Congo, illegal tree logging represents over 75% of the annual industrial timber production (Nellemann 2012). DNA-based identification tools can support investigations into illegal trade, but depend upon an accurate genetic reference database to identify and trace the provenance of logged trees. In this regard, herbarium collections are an excellent source to generate such genetic reference databases, especially in areas where field expeditions are not feasible anymore due to political instability or increased inaccessibility. Here, we demonstrate the usefulness of genome skimming by shotgun sequencing to mine herbarium specimens for the assembly of their plastomes to support DNA-based identification of trade timber species.

The quality and quantity of DNA in herbarium specimens is strongly reliant on collection and storage conditions and, in general, herbarium DNA can be highly fragmented (< 150 bp) and only available in very low amounts (< 5 ng/µl). Interestingly, the techniques optimised for historical herbarium specimens can also be applied to heartwood specimens (i.e. old degenerated material) of processed wood. By jointly analysing herbarium material and silica-dried leaf samples, a clear comparison can be made on the feasibility of historical material for genome skimming purposes, with the aim of yielding full plastid genomes of selected species that are under strong pressure due to illegal logging activities in Central Africa.

### Materials and Methods

In order to obtain plastomes of the most important timber species from equatorial African tree species, we collected leaf tissue samples (2 cm^2^; ca. 10 mg) from 12 herbarium specimens and 20 silica samples via various herbaria (BR, BRLU & L; Suppl. material 3). Tree species were selected, based on the following criteria: providing highly valuable timber, becoming potentially important for national and international timber trade or for being reported in agreements on global biodiversity conservation (e.g. CITES, IUCN). In the case of herbarium samples, there was a careful selection based on prior knowledge about the specimens. We avoided material that was likely to have been: (a) dried with alcohol, (b) treated with conservatives posterior to collection (e.g. HgCl_2_) or (c) collected in remote areas where it was difficult to properly dry the specimens in the field. When sampling from herbarium specimens, we aimed to: (d) collect the greenest leaf tissue, (e) avoid the central leaf vein and (f) avoid leaves with potential markings of insect herbivory.

**DNA extraction and library preparation**

Total genomic DNA of both silica and herbarium material was extracted using a combined and modified version of the CTAB protocol (Doyle and Doyle 1987) in which an initial washing step with 0.35 M d-sorbitol was included. The lysis buffer contained 2% CTAB and 2% PVP-40. A chloroform-isoamyl alcohol (24/1 v/v) extraction was carried out twice. After a cold isopropanol precipitation and subsequent centrifugation, the pellet was washed with 70% ethanol and air-dried. The DNA pellet was eluted with 1X TE buffer. All herbarium specimen DNA extractions were carried out under a laminar flow hood, in which positive air pressure and UV disinfection was present.

The purity of the resulting DNA was measured under the absorbance ratios OD 260/280 and OD 260/230 using a NanoDrop 2000 (Thermo Fisher Scientific, US). DNA concentration (ng/μl) and fragment size distribution were measured by capillary electrophoresis using a Fragment Analyzer (Agilent, US). Library preparation of the silica-dried leaf material was initiated via an enzymatic DNA fragmentation step with the aim to retain DNA fragments with a size between 200 and 450 bp after which an end repair step took place. This step was conducted using the NEBnext UltraTM II FS DNA Library Prep Kit for Illumina (New England Biolabs, US). Due to the presence of already degraded DNA in the herbarium specimens, the enzymatic DNA fragmentation step was not carried out for the herbarium material. Adapter ligation was conducted using the NEB-next Adaptor kit for Illumina and U-excision was carried out using the USER Enzyme kit (New England Biolabs, US). Size selection (320–470 bp) was conducted following the SPRIselect protocol (Beckman Coulter, US). With the NEBNext Ultra II Q5 Master Mix, adaptor-ligated DNA was indexed, then PCR-enriched with the NEBNext Multiplex Oligos for Illumina (New England Biolabs, US). For the latter, the following thermocycler reactions were used: Initial denaturation at 98°C for 30 s, 3–4 cycles of denaturation at 98°C, each for 10 s as well as an annealing/extension at 65°C for 75 s and a final extension phase at 65°C for 5 min. In the last step of the library preparation, a DNA-library purification was conducted using SPRIselect (Beckman Coulter, US). The final fragment size distribution and molarity (nM) were examined with a Fragment Analyzer (Agilent, US). Indexed libraries were subsequently pooled (on average 25 samples per lane) in equimolar ratios. Sequencing of the DNA libraries (low coverage paired-end; 10X, 150 bp) was done on a HiSeq 3000, a HiSeq 4000 and NovaSeq 6000 (Illumina, US). At the time of analysis, between autumn 2019 and spring 2021, library preparation and sequencing costs were estimated at a total of €45 - 50 per sample.

**Data analysis**

The quality of the raw reads was evaluated with FastQC (Andrews 2010). Using the GetOrganelle pipeline, plastomes were de-novo assembled (Jin et al. 2020) under *k-mer* values set as “-k 21, 45, 65, 85, 105” for all species and 15 extension rounds. The pipeline was initiated by recruiting targeted plastid-like reads as applied in Bowtie 2 (Langmead and Salzberg 2012). During the assembly process, reads were trimmed and contigs reconstructed with SPAdes 3.13 (Bankevich et al. 2012). In addition, plastid-like contigs were filtered by comparing them against the BLAST nucleotide database following the NCBI Blast+ tool (Camacho et al. 2009). In the next step, reconstructed plastomes were aligned against a reference genome with MAFFT v.7 (Katoh et al. 2002), thereby aiming for the most closely-related taxon for comparison that could be found on GenBank. In the case of unsuccessfully assembled plastome regions using the GetOrganelle pipeline, raw reads were then mapped to target regions of closely-related species using Bowtie 2 (*Guibourtiapellegriniana* and *G.tessmannii*). Applying the web-based software CpGAVAS2 (Shi et al. 2019), annotation was conducted, after which the annotation results were endorsed with Geneious Prime (Kearse et al. 2012) by comparing them with a reference plastome derived from GenBank.

### Results and Discussion

Amongst the 16 herbarium specimens, DNA yields varied between 220 and 430 ng/µl, whereas DNA yields of silica-dried samples varied between 210 and 850 ng/µl (based on starting leaf tissue samples of 2 cm^2^). All taxa investigated yielded sufficient DNA and were used for library preparation and sequencing. For the herbarium specimens, between 200,000 and 5.2 million high-quality paired-end reads were produced, whereas for the silica-dried samples, this amount varied between 1.6 million and 6.4 million reads. Over 4 million high-quality paired-end reads were retrieved for only 19% of the herbarium specimens, whereas for the silica-dried samples, there was a ca. 50/50 split of accessions above or below 4 million high-quality paired-end reads. Genome coverage depth was at least 50x for all specimens with fully assembled plastid genomes. Even though a very small number of reads were retrieved from some specimens, it was possible to generate complete plastomes for the majority of the herbarium specimens (Mascarello et al. 2021). The complete plastomes always consisted of a small circular sequence partitioned in four main structures that are typical for land plants: a large single copy region (LSC) and a small single copy region (SSC), disconnected from each other by two inverted repeats (IRa and IRb). There was only one specimen where the number of reads was below 1 million from which a full plastome could not be obtained. Moreover, for this accession, the percentage of duplicate reads was 5%, whereas for all other accessions, the percentage of duplicate reads varied between 9 and 13%. The quality of the plastome sequence was checked by translating all gene regions. No stop codons (indicative of sequencing errors) were observed along the assembled contigs. Using this approach, a genetic reference database of threatened African trees has been developed as a tool against illegal logging, as well as an optimised DNA isolation protocol to obtain sufficient DNA via the Genome Skimming by Shotgun Sequencing method (Cronn et al. 2008, Mascarello et al. 2021).

The results obtained in this case study corroborate those of some recently-published studies on the use of genome skimming for the retrieval of full plastomes of land plants (Bakker et al. 2016, Zeng et al. 2018, Alsos et al. 2020, Nevill et al. 2020). Each of those studies indicates the scalable potential of genome skimming to obtain plastome sequences from herbarium specimens. Using this approach, a high level of success has been achieved across a range of ages of herbarium specimens (Alsos et al. 2020, Nevill et al. 2020) and even with small amounts of tissue, an effective plastome assembly can be generated (Bakker et al. 2016). This collective body of studies shows that genome skimming represents an inexpensive, pragmatic approach for recovery of plastome sequences that can be applied to small amounts of herbarium material.

*Main findings and recommendations*:

- Genome skimming of herbarium specimens has shown high success rates across multiple independent studies;
- Despite the often lower number of reads retrieved from herbarium specimens compared to fresh tissue, it is becoming increasingly routine to generate complete (or almost complete) plastomes from herbarium material using genome skimming;
- Since one of the most important steps in the genome skimming protocol is to downsize the DNA fragment length, the often highly-degraded DNA of herbarium specimens allows the sonication step to be bypassed in the library preparation protocol.

*Data availability*: The data in this case study are available under the following GenBank numbers: MZ274087, MZ274092, MZ274094-MZ274096, MZ274099, MZ274102-MZ274107, MZ274110, MZ274113, MZ274116-MZ274122, MZ274124, MZ274127-MZ274129, MZ274132, MZ274135, MZ274137, MZ274143, MZ274145, MZ274147, MZ274148 (see Mascarello et al. (2021)).

## Case study 4: Comparing hybridisation capture derived sequences from herbarium specimens with data from living material of the same genetic individuals

### Introduction

Herbarium collections worldwide contain an estimated 350 million specimens collected over the last approximately 400 years (Besnard et al. 2018). Advances in DNA extraction and sequencing technologies are making herbarium specimens increasingly accessible to retrospective genomic analyses. Sequencing approaches include shotgun metagenomics (Bieker et al. 2020), Whole Genome Sequencing (WGS) (Yoshida et al. 2013), genome skimming (Bakker et al. 2016, Zeng et al. 2018, Nevill et al. 2020, Case study 3) and hybridisation capture (Hart et al. 2016, Sánchez Barreiro et al. 2017, Gutaker et al. 2019).

The preservation and quality of DNA in herbarium material are highly variable. It has been suggested that DNA decays at a faster rate in plant remains compared to animals (Allentoft et al. 2012, Weiß et al. 2016) and the techniques used for herbarium sheet preparation and the storage conditions of specimens have been shown to affect DNA recovery and fragmentation rates (Särkinen et al. 2012, Forrest et al. 2019). Studies comparing recently prepared and older herbarium specimens do not reach a consensus on whether DNA fragmentation and damage happen mainly at specimen preparation (e.g. Staats et al. (2011)) or accumulate gradually over time (e.g. Weiß et al. (2016)). These discrepancies are likely the result of different preparation techniques, ranging from gentle drying in non-acidic paper to high heat and chemical treatments. For this reason, for herbarium specimens, it is common to see both laboratory protocols aimed at recovering and sequencing low-concentration, fragmented DNA (e.g. Latorre et al. (2020)) and protocols commonly used for higher-quality DNA sources (e.g. Forrest et al. (2019)).

In this study, we sampled specimens from the herbarium at the Royal Botanic Garden Edinburgh (RBGE) that were collected 12-50 years ago from cultivated individuals of *Rhododendronjavanicum*. These cultivated individuals are still present as live plants in the living collection at RBGE and allowed us to assess the reliability of sequences recovered from herbarium material compared to freshly-collected samples from the same genetic individuals. The chosen sequencing approach was hybridisation capture (also known as target capture or target DNA enrichment) which is an effective sequencing approach for studies utilising degraded DNA sources because it enables recovery of sequence data from low concentrations of endogenous DNA (Carpenter et al. 2013).

### Materials and Methods

**Samples**

Twelve RBGE herbarium vouchers (dated 1972-2010) of various sub-species of cultivated *Rhododendronjavanicum* were sampled along with fresh leaf material from 10 living individuals growing in the RBGE glasshouses, from which the herbarium vouchers were generated. Two of the living individuals were represented by two separate vouchers, collected one or ten years apart. Herbarium samples and their corresponding living samples are listed in Table 4. Additional sample information is provided in Suppl. material 4.

**DNA extraction and library preparation - herbarium samples**

DNA from herbarium specimens was extracted as described in Latorre et al. (2020), using the Basic Protocol 1, following standard anti-contamination precautions (Gilbert et al. 2005, Llamas et al. 2017), including parallel non-template controls. DNA fragment size distribution for extracts was inspected with the gDNA Kit on the Agilent Femto Pulse System. Sequencing library preparation protocol was selected based on DNA fragment size (Table 4).

DNA extracts with fragments shorter than 500 bp (n = 5) were converted into single-stranded DNA (ssDNA) libraries following Kapp et al. (2021) with tier four adapter dilutions and unique dual indexes. All steps up to indexing PCR reactions were carried out in dedicated ancient DNA facilities at the University of Oslo, Norway. Sequencing library quality and concentration were assessed by qPCR following Meyer and Kircher (2010) and with the Ultra Sensitivity NGS Kit on the Agilent Femto Pulse System. Libraries were then re-amplified in three 25 μl reactions with Herculase II Fusion DNA polymerase (Agilent). One sample (RHD011) with longer DNA fragments was also included in this library preparation batch.

Of the remaining samples (n = 9), we recovered less-fragmented DNA, including samples with a bimodal DNA fragment size distribution (n = 7), with one peak of fragments shorter than 1000 bp and a second peak of approximately 1-20 kbp; the other samples included one sample (RHD005) with DNA fragments of 100-1000 bp and one (RHD018) with mostly short fragments, but with a tail of longer fragments. Aliquots of these extracts were subjected to 8-12 sonication cycles of 30 s on, 90 s off, using a Diagenode Bioruptor sonicator, for a target fragment size of 200-400 bp. Libraries were generated with the NEBNext® Ultra™ II DNA Library Prep Kit for Illumina (New England Biolabs) and indexed with NEBNext® Multiplex Oligos for Illumina® (Unique Dual Index Primer Pairs). Because these libraries were produced in non-dedicated facilities where fresh plant material is regularly processed — posing a risk for contamination — the following anti-contamination precautions were taken: pre-amplification steps were carried out inside a dedicated laminar flow hood in a pre-PCR room with dedicated reagents and consumables and negative non-template controls were included.

**DNA extraction and library preparation - living collection samples**

Approximately 150 mg of leaf material was harvested into 7.6 ml FluidX tubes and placed immediately into liquid nitrogen. DNA was extracted using a protocol developed for extracting high molecular weight DNA (Nishii et al. 2023). This protocol, which includes a sorbitol wash prior to using the Qiagen Genomic Tip Kit, was used due to the high quantity of secondary metabolic compounds present in *Rhododendron*. The DNA extracts were sonicated for 7-11 cycles of 30 s on, 90 s off, using a Diagenode Bioruptor sonicator, for a target fragment size of 200-400 bp. Libraries were generated with the NEBNext® Ultra™ II DNA Library Prep Kit for Illumina (New England Biolabs) and indexed with NEBNext® Multiplex Oligos for Illumina® (Unique Dual Index Primer Pairs). These libraries were generated in non-dedicated facilities with pre-amplification steps carried out inside a dedicated laminar flow hood with dedicated reagents and consumables.

**Hybridisation capture and sequencing**

Hybridisation capture was performed on all libraries. The assay was designed using two published *Rhododendron* genomes from NCBI: *R.delavayi* (Zhang et al. 2017) and *R.williamsianum* (Soza et al. 2019) and a transcriptome from the mature leaf of *R.scopulorum* from the 1000 Plants (1KP) project (Matasci et al. 2014). The bait set contains 492 target loci, including 298 loci orthologous to the Angiosperm353 universal probe set (Johnson et al. 2019). The remaining 194 loci were picked from genes related to cold tolerance, flowering pathway, key developmental regulators of meristem function, organ development and trichome development. Baits were synthesised by MyBaits (Arbor Biosciences) with 3X bait tiling to be optimal for degraded DNA.

Libraries were pooled according to material and library construction protocol. The samples were processed with a wider set of samples than are presented here, such that each pool contained 10-14 libraries. Negativecontrols were pooled separately. Hybridisation capture was performed following the MyBaits (Arbor Biosciences) protocol v.5.02 with the high sensitivity version and the hybridisation and wash temperatures set to 63°C for herbarium samples (the second round of enrichment was omitted) and with the standard version and hybridisation and wash temperatures set to 65°C for living samples. Pools were re-amplified post-capture in two 50 μl reactions with Herculase II Fusion DNA polymerase (Agilent) for 14 cycles. Captured libraries for living and herbarium samples were sequenced on separate Illumina MiSeq lanes with no index repetition, with 150 bp PE v.2 runs (4.5 - 5.0 Gb) at the University of Exeter sequencing facilities. The target region of the bait set is 621,078 bp, which translates to an average coverage of 720X/sample for the 10 living samples and 600X for the 12 herbarium samples.

**Data analysis**

Herbarium reads were processed with the PALEOMIX v.1.3.7 BAM pipeline (Schubert et al. 2014). Paired-end reads were trimmed, filtered, and collapsed with AdapterRemoval v.2.3.3 (Lindgreen 2012), discarding reads shorter than 25 bp. Collapsed reads were aligned to the target loci used for probe design with BWA v.0.7.17 (Li and Durbin 2009), using the backtrack algorithm with disabled seeding and a minimum quality score of 25. mapDamage v.2.2.1 (Jónsson et al. 2013) was used to assess aDNA deamination patterns and rescale BAM file quality scores. Living collection reads were processed as described for herbarium reads without read collapsing and retaining reads longer than 50 bp. The BWA MEM algorithm was used for read alignments to the same references.

Quantity and quality of the SNPs called for the herbarium samples were assessed by comparison to the sequence from their respective paired living sample. First, a new reference for each individual was generated using sequence data from only the living sample of that individual. BAM files from the initial run of PALEOMIX (above) were filtered using strict settings on bcftools v.1.16 (filter SNPs by QUAL > 160 and DP > 10) and consensus fasta files were generated to be used as a new reference (Forrest et al. 2019). The new reference was used to run the PALEOMIX BAM pipeline for a second round for the same living and their respective herbarium sample pairs, this time using the individual new references rather than the original target sequences. New VCF files were generated from the output BAM files and bcftools stats was used to compare SNPs called from the living and from the herbarium material. We identified shared SNPs in living and herbarium samples, but not present in the new reference (likely heterozygous sites) and those exclusive to the herbarium samples (likely erroneous SNPs). The code used to analyse the data and make figures is available at: https://github.com/rbgedinburgh/dna_sequencing_herbaria.

### Results and Discussion

**Evaluating sequencing library preparation for herbarium material and contamination control**

**Figure 6.**
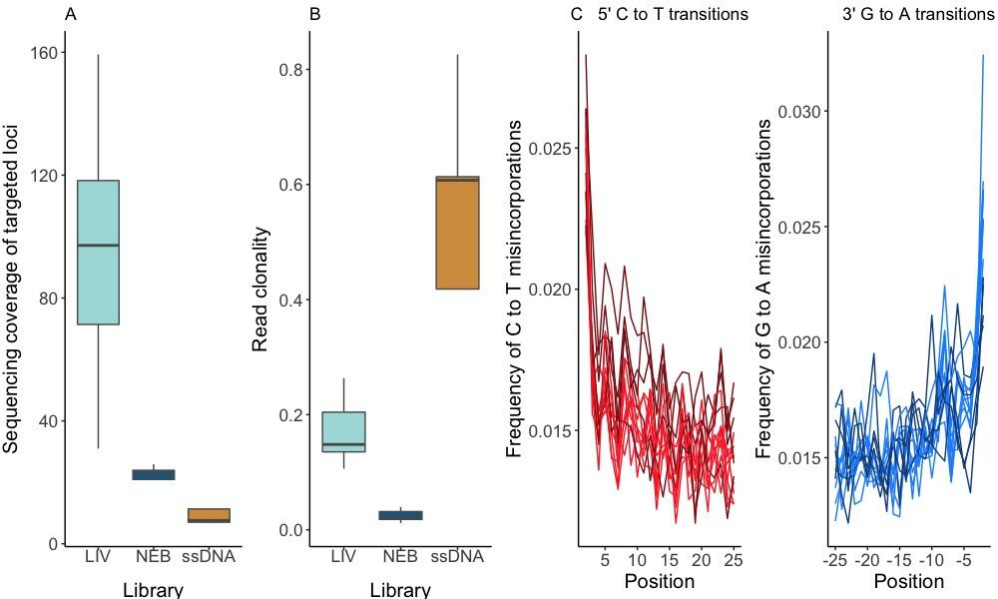
Sequencing coverage of targeted loci and library complexity for *Rhododendron* specimens. **A** Coverage of targeted nuclear loci. **B** Proportion of PCR duplicates. LIV = libraries generated from living collection samples, ssDNA = single-stranded DNA libraries made from degraded herbarium DNA, NEB = double-stranded DNA libraries made from sheared herbarium DNA using a commercial kit. **C** DNA deamination patterns of read data obtained from NEB (red, blue) and ssDNA (dark red, dark blue) herbarium libraries with mapDamage v.2.2.1. First base was removed for visualisation.

Without any prior assumption of DNA fragmentation rates in the herbarium samples processed in this study, our approach consisted of isolating DNA in dedicated clean facilities. Following an assessment of DNA fragment size, we decided to separate samples into two categories. Fragmented DNA extracts were treated, following aDNA protocols in dedicated facilities, whereas extracts containing longer DNA fragments were taken to non-dedicated facilities for DNA shearing and library preparation using commercially available kits. We assessed coverage of targeted loci (Fig. 6A) and library complexity —using read clonality — (Fig. 6B) by mapping reads to the target loci used for probe design. As expected, we obtained higher coverage of targeted loci from freshly-collected samples for similar amounts of sequencing effort. For herbarium samples, libraries generated from sheared DNA using the NEB kit had higher complexity and higher coverage of targeted loci than ssDNA libraries that were generated from highly-degraded DNA. This is to be expected given the difference in quality of input DNA. Detailed mapping statistics are available in Suppl. material 5.

We did not detect any amplification products in the negative controls of libraries generated in the non-dedicated facilities following anti-contamination measures, indicating that it was possible to process herbarium DNA extracts of sufficient DNA concentration and fragment size under these conditions and with the necessary precautions. In addition to the ssDNA library construction protocol (Kapp et al. 2021), we also tested a dsDNA protocol optimised for aDNA (Meyer and Kircher 2010, Kircher et al. 2012) (data not shown), but we observed high levels of sequence clonality, possibly caused by PCR inhibition. DNA isolation and sequencing library preparation for plant material can be complicated by secondary compounds, such as polysaccharides and polyphenols that can bind to and co-precipitate with DNA, resulting in PCR-inhibition (Souza et al. 2012). *Rhododendron* is rich in secondary metabolic compounds (which also led to difficulties in extracting DNA from fresh material) and it is possible that the initial DNA denaturation step in the ssDNA library preparation protocol had a beneficial effect on breaking crosslinks between DNA and secondary compounds (compared to the dsDNA protocol). We only tested a small number of samples, but the efficacy of this comparatively fast and cheap ssDNA protocol is promising and further testing on short degraded DNA isolated from herbarium material would be worthwhile.

Finally, we observed mild deamination patterns in reads recovered from herbarium material (Fig. 6C) compatible with historic DNA damage (Jónsson et al. 2013), although the magnitude of this was very small (ca. 3% first base misincorporation) compared to levels often observed in older material. Interestingly, we observed similar deamination patterns in libraries generated with the ssDNA protocol and the NEB kit, indicating that despite DNA shearing, and the NEB library preparation including a USER enzyme hairpin loop adaptor cleavage step, enough base deaminations at DNA overhangs were retained to show evidence of post-mortem DNA damage (Jónsson et al. 2013).

**Assessing reliability of SNPs recovered from herbarium material**

We took advantage of cultivated plants present in the RBGE living collection, from which herbarium vouchers were created 12-50 years ago, to investigate whether sequences recovered from the herbarium samples were an accurate biological replicate of the living material, or if low starting templates and base modifications, both features that accumulate in degrading DNA over time, resulted in erroneously-called bases (Briggs et al. 2007). Using only sequences recovered from living material, we assembled a strict consensus sequence for each individual. These were used as a new reference for mapping and SNP calling. We assigned SNPs as being exclusive to living samples, exclusive to herbarium samples or shared between a living-herbarium pair of the same individual. SNPs exclusive to living samples might be caused by ambiguous calls at heterozygous sites, while SNPs exclusive to herbarium samples can be interpreted as erroneous SNPs, likely due to low SNP quality, low coverage or base modifications in degraded DNA. In contrast, shared SNPs between living and herbarium tissue can be interpreted as true.

**Figure 7.**
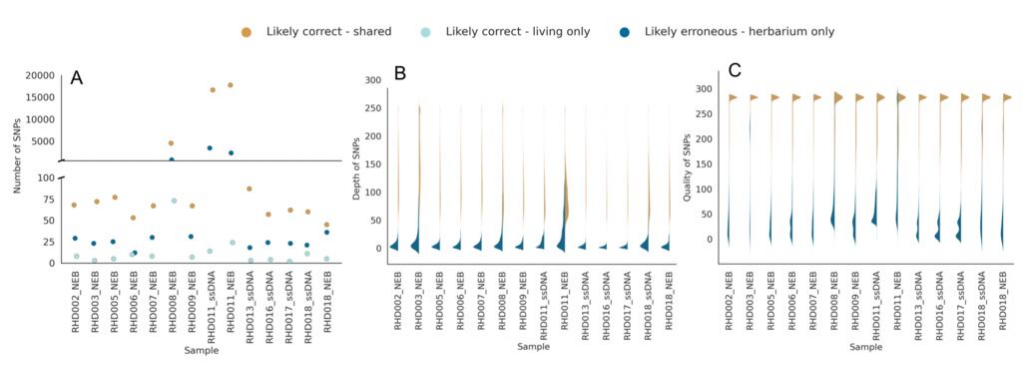
Comparison of SNPs recovered from herbarium and living collection samples of the same individuals of *Rhododendron* species. **A** Number of SNPs called, categorised into exclusive to living samples (light blue, likely caused by ambiguous calls at heterozygous sites), exclusive to herbarium samples (dark blue, likely caused by sequencing errors due to degraded DNA) or shared (yellow). **B** Depth and **C** quality of shared and herbarium-exclusive SNPs. ssDNA = single-stranded DNA libraries made from degraded herbarium DNA, NEB = double-stranded DNA libraries made from sheared herbarium DNA using a commercial kit.

We typically observed 75-108 SNPs per individual, of which 45-87 were shared between living and herbarium samples (Fig. 7A) and 12-36 that were found in herbarium specimens only. We did not observe a correlation between specimen age and proportion of these likely erroneous SNPs. We also inspected the quality and sequencing depth of SNPs and found that SNPs exclusive to herbarium samples were of much lower quality than those present in both herbarium and living material (Fig. 7B and C). The quality and depth of these erroneous SNPs unique to herbarium specimens are, however, above standard SNP-filtering thresholds. In our study, the use of more stringent filtering criteria for herbarium SNPs is required to give a better representation of ‘true’ sequence variants (e.g. those also recovered from non-degraded tissues).

Two samples (represented by three libraries) showed a noticeable spike in SNP abundance compared to all others. Both of these samples are from the same sub-species Rhododendronjavanicumssp.palawanense (RHD008 and RHD011) and retrospective analyses of their morphology suggest they may be of hybrid origin. It is possible that the observed spike in SNP abundance is due to these specimens having higher levels of heterozygosity due to hybridity. With a greater number of variable sites, there is an associated increased possibility of detecting both genuine (shared) SNPs with respect to the reference, as well as a corresponding increase in erroneous SNPs due to poor coverage of these sites in herbarium material.

**Implications for sequencing herbarium specimens**

Multiple studies have now been undertaken exploring the potential of hybridisation capture for the recovery of sequence data from herbarium specimens. These have included exploratory studies assessing the feasibility of the approach for recovering sequence data from plant specimens with a range of different ages (Hart et al. 2016), studies evaluating the impacts of different treatment methods on sequencing success (Brewer et al. 2019, Forrest et al. 2019) and those exploring the practicalities of scaling hybridisation capture in plants, including how the characteristics of specimen origin and condition influence sequence recovery (Folk et al. 2021, Kates et al. 2021). Collectively, these and other studies have provided clear evidence that the recovery of large amounts of nuclear sequence data is feasible for herbarium specimens with a wide range of ages and across different taxonomic groups. In the current case study, we have shown that erroneous base-calls can be made due to low starting templates and modified bases in herbarium specimens. However, with stringent filtering for quality and depth, these erroneous SNPs can be excluded, such that the remaining SNPs represent a more accurate reflection of the individual’s genotype.

*Main findings and r ecommendations*:

- Hybridisation capture is now well established as a method for recovery of large amounts of nuclear sequence data from herbarium specimens and the approach works well in accommodating the complexity of plant genomes;
- Studies recovering DNA from herbarium specimens should take place in dedicated clean or low-copy facilities. Once DNA fragment length distribution is known, sequencing library preparation can take place according to DNA size;
- Library preparation from highly-fragmented DNA should take place in dedicated clean or low-copy facilities. We found the ssDNA protocol by Kapp et al. (2021) to be fast and efficient for this purpose in the current study and the simplicity of this approach warrants further trialling to see if these results are generally applicable;
- DNA extracts that show a bimodal fragment size distribution with the majority of fragments > 1 kb can be sheared, prior to library preparation with a commercially-available kit. If this takes place in non-dedicated facilities, we recommend the following contamination-limiting precautions:
  - Physical separation of pre- and post-PCR laboratories;
  - Dedicated laminar flow hood for all pre-PCR steps (to be regularly decontaminated);
  - Dedicated reagents and consumables;
  - Inclusion of non-template negative controls;
- Distribution of SNP quality and coverage should be inspected for a better-informed decision on filtering parameters. Stringent quality filtering of SNPs can provide high confidence in genotype calls even from herbarium material.

*Data availability*:

The raw sequencing data has been deposited at the European Nucleotide Archive (ENA) under project PRJEB61704 with accession IDs ERS15567903 - ERS15567924.

## Case study 5: Selecting samples with the greatest likelihood of success for reduced-representation sequencing from museum collections

### Introduction

Reduced-representation sequencing (RRS) using restriction digests followed by fragment sequencing is a cost-effective route for generating thousands of genetic markers (Davey and Blaxter 2010, Davey et al. 2011, Puritz et al. 2014). Although this type of approach has proved very effective when working with high-quality DNA (Nadeau et al. 2014, Van Belleghem et al. 2018), its implementation in museum studies has been hampered by the unpredictable outcomes due to DNA degradation of museum specimens (Graham et al. 2015, Souza et al. 2017, Lang et al. 2020). DNA degradation at restriction sites causes failure or bias in RRS due to inefficient or failed restriction digests, while random shearing lowers the number of fragments being flanked by both restriction sites and therefore prevents adapter ligation (Puritz et al. 2014, Graham et al. 2015). A study on artificially-induced DNA degradation illustrated a significant decrease in the number of RADtags per individual, the number of variable sites and the percentage of identical RADtags retained (Graham et al. 2015). These difficulties have dissuaded scientists from using RRS as a tool to obtain museum population-level data. However, when large collections are available, a careful screening assessment prior to library preparation could aid in the selection of samples that are most likely to yield successful results. Therefore, we here assess: (i) to what extent DNA degradation affects the success rate of RRS in a long-term time series of avian museum specimens and (ii) whether we can predict *a priori* the success rate of RRS on museum samples using easy-to-obtain DNA quality metrics.

### Materials and Methods

**Sampling**

We sampled 96 barn owls (*Tytoalbaalba*) comprising both historical as well as contemporary specimens. Historical samples were obtained from collections stored at the Royal Belgian Institute of Natural Sciences and covered two distinct periods in time, mainly from the 1930s (1929-1943, n = 15) and mainly from the 1970s (1966-1979, n = 22). Contemporary specimens (n = 59) comprised road kills which were brought to bird sanctuaries and stored in freezers immediately upon arrival. We collected toe pads of all historical specimens to minimise voucher damage and liver or breast muscle tissue of the contemporary specimens.

**DNA extraction, library preparation and SNP calling**

DNA of all specimens was extracted using the NucleoSpin Tissue Kit (Macherey-Nagel GmbH). Concentrations were quantified by the Qubit fluorometer (Invitrogen) and a fragment analysis of historical samples was conducted on a 2100 Bioanalyzer (Agilent). While numerous variations on reduced-representation genome sequencing exist (Puritz et al. 2014), we here focused on double-digest restriction site-associated DNA sequencing (ddRAD) because of the simplified wet-lab workflow, low cost and highly-homogenous coverage of sites across samples (Peterson et al. 2012). ddRAD libraries were constructed following the protocol of Peterson et al. (2012). Briefly, we digested DNA samples using two restriction enzymes, i.e. SbfI and MseI. Starting volumes of DNA were adjusted according to sample specific DNA concentrations (18 µl, 12 µl or 6 µl of DNA when concentrations were respectively lower than 20 ng/µl, between 20 and 32 ng/µl or higher than 32 ng/µl). Barcoded SbfI and universal MseI-compatible adapters were subsequently ligated to the digested genome, followed by a size selection of fragments of 270 bp (“narrow peak” setting) on a BluePippin (Sage Science). Lastly, fragments were PCR amplified using a barcoded reverse primer to obtain dual-indexed ddRAD libraries, which were subsequently paired-end sequenced on an Illumina Novaseq6000 platform. Raw data were demultiplexed using the process_radtags module in Stacks v.2.50 (Catchen et al. 2011). Trimmomatic v.0.39 (Bolger et al. 2014) was used to remove adapters and a sliding window approach was applied to trim reads when quality fell below 20. Paired reads were mapped to a reference genome (GCA_000687205.1_ASM68720v1) using BWA mem (Li and Durbin 2009) using default settings and only properly paired reads with a quality > 30 were retained using SAMtools v.1.11 (Li et al. 2009). SNPs were subsequently called using GATK’s HaplotypeCaller tool (McKenna et al. 2010).

**Contamination assessment**

In order to avoid any bias in downstream analyses arising from contaminated historical specimens, we first assembled a stringently-filtered vcf based exclusively on recent samples. Specimens showing more than 20% missing data were discarded and only biallelic SNPs (--max-alleles 2) with a minimal SNP quality (--minQ) of 40 and an individual genotype (--minGQ) quality of 30, present in at least 50% of the individuals (--max-missing) and a minimum allele frequency (--maf) of 0.01 were retained with VCFtools (Danecek et al. 2011). This resulted in a dataset of 31,012 SNPs covering 10,349 RADtags and 0.3% of the genome. These reference SNPs were then subsequently extracted from all individuals, for example, both historical as well as contemporary specimens, to limit the erroneous inclusion of exogenous DNA sequences from historical samples. As the SNP discovery protocol is exclusively applied on recent samples, this could, however, result in a SNP ascertainment bias and concomitant underestimation of genetic diversity in historical populations or erroneous measures of genetic differentiation (Lachance and Tishkoff 2013). To eliminate such bias, one should identify a sufficient number of high-quality historical samples with minimal missing data and repopulate the SNP discovery pipeline with this extended dataset.

**Statistical analysis**

We ran a one-way ANOVA to test for differences in mean number of missing SNPs amongst the three time periods and allowed for period-specific variances to account for heteroscedasticity using the R package ‘nlme’ (Pinheiro et al. 2022). To predict the success rate of ddRAD in museum samples, we applied generalised additive models (GAM) to relate the percentage of missing SNPs per individual to either DNA concentration or fragmentation using the R package ‘mgcv’ (Wood 2011). All statistical analyses were performed using the R 4.1.2. software (R Core Team 2021). DNA fragmentation was assessed from Bioanalyzer profiles by calculating the percentage of the area under the curve in four distinct bins, i.e. bins that contain fragments ranging from, respectively, 35-200 bp, 200-400 bp, 400-700 bp or 700-10380 bp.

### Results and Discussion

**Figure 8.**
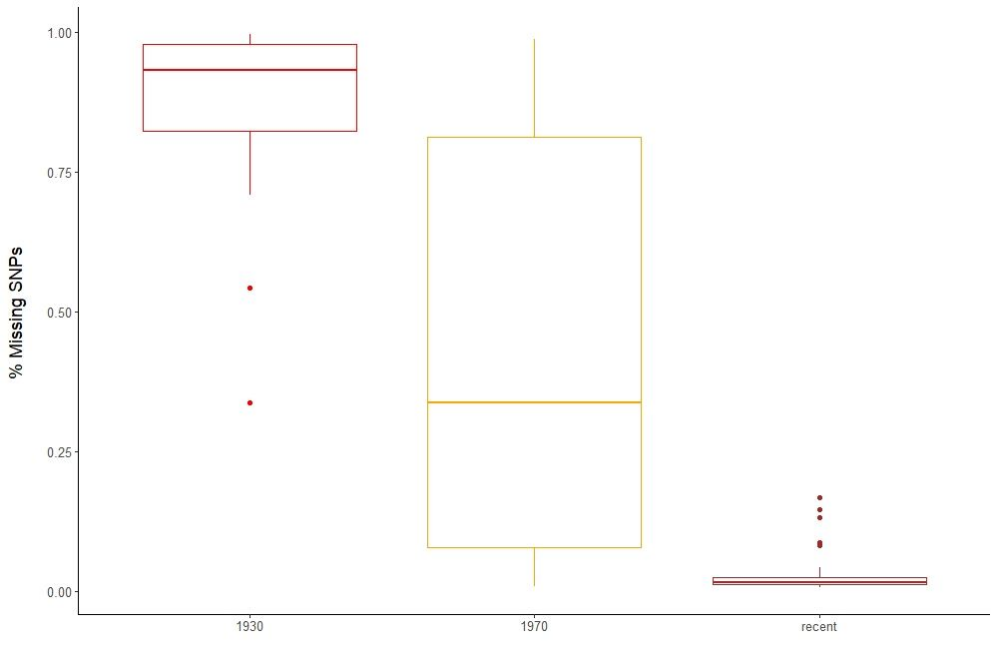
Percentage of missing SNP data per individual of *Tytoalbaalba* in museum specimens of different ages and recently collected material.

Mean missing data per individual differed significantly between time periods (chi-square = 62.56, p < 0.001) (Fig. 8). The mean percentage of missing SNPs was 2.6% for recent specimens, 43.4% for specimens sampled around the 1970s and 85.4% for specimens originating from around the 1930s. The variance in missing data varied significantly between time periods (Breusch-Pagan test, chi-square = 52.1, p < 0.001). Recent samples showed consistently few missing SNPs, while the success rate in samples from the 1930s varied slightly more. In contrast, samples from the 1970s showed large variation in missing data, ranging from highly-successful samples to those that failed almost completely, complicating the utility of age of the sample as a suitable predictor for success of RRS of museum specimens.

**Figure 9.**
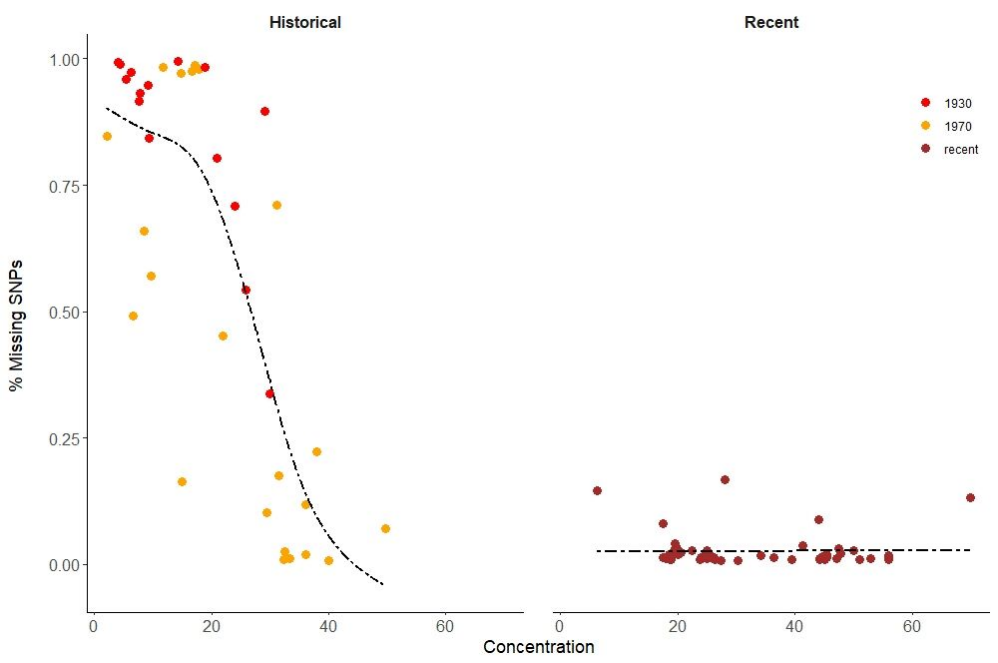
The association between DNA concentration and percentage of missing SNPs in historical and contemporary samples of *Tytoalbaalba*. The dashed line represents the predicted values according to the fitted GAM (historical samples) and linear regression (recent samples).

Mean DNA concentration in historical and recent samples were respectively 20.2 ng/µl ± 12.4 (SD) and 30.6 ng/µl ± 13.9 (SD). A simple linear regression indicated the number of missing SNPs was not related to DNA concentration in recent samples (F_1,57_ = 0.016, p = 0.90). In contrast, a GAM indicated DNA concentration was inversely related to the amount of missing data in historical samples (F_1,3.2_ = 15.97, p < 0.001) and explained 66.8% of the deviance (Fig. 9).

**Figure 10.**
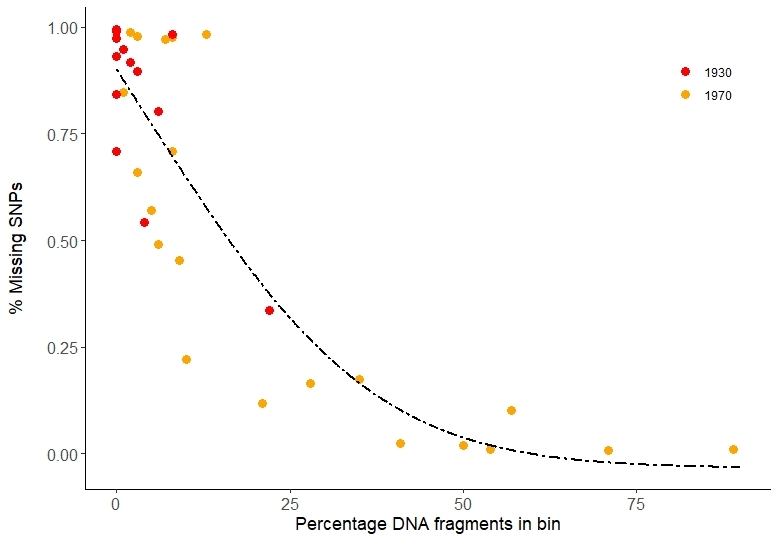
Inverse association between the percentage of DNA fragments in the highest bin range (700-10380 bp) and percentage of missing SNPs in historical *Tytoalbaalba* samples. The dashed line represents the predicted values according to the fitted GAM.

GAMs relating the amount of missing data to the percentage of fragments between 35-200 bp, 200-400 bp, 400-700 bp and 700-10,380 bp explained, respectively, 74.8%, 20.7%, 39.7% and 78.4% of the model deviance. The amount of fragments in the lowest bin range was strongly positively associated with the levels of missing data (F_1,2.3_ = 32.99, p < 0.001), while those at the highest bin range showed a clear negative association (F_1,2.4_ = 37.63, p < 0.001) (Fig. 10). Based on the latter model, the predicted amount of missing data when 1%, 5%, 10%, 20%, 30% or 50% of fragments ranged between 700 bp and 10,380 bp was, respectively, 88%, 77%, 65%, 42%, 23% and 4%, clearly illustrating the association between missing data and fragmentation.

To date, few studies have assessed whether RRS on museum collections is feasible, and if so, how to optimise approaches. In a previous study using ddRAD target enriched sequencing, an inclusion threshold for DNA concentration of 30 ng/µl was suggested (as determined from the A260 values) (Souza et al. 2017). A similar finding emerges from our study, as the percentage of missing data was notably lower from samples with DNA extract concentrations above 30 ng/µl (Fig. 9). However, overall we note that DNA fragmentation was a better predictor for the percentage of missing SNPs and successful sequencing, compared to DNA concentration. DNA concentration was not always perfectly inversely associated with DNA fragmentation as some samples with low DNA concentration also showed low levels of DNA fragmentation, or conversely, some samples with high DNA concentration were highly fragmented. Furthermore, DNA concentration of problematic samples can be increased by eluting in smaller volumes or lysing more tissue during DNA extractions, yet, fragmentation profiles will still remain unaffected. Lastly, unlike fragmentation profiles, sample DNA concentrations are species and tissue dependent, making it difficult to set a universal threshold.

ddRAD appears unsuitable to obtain sequence data from highly-fragmented samples (in our case, the older museum samples dating from the 1930s and some more recently collected material from the 1970s). More advanced target-capture-based technologies such as HyRAD and HyRAD-X should be considered as an alternative (Suchan et al. 2016, Schmid et al. 2017), although these technologies do require additional steps and higher costs. However, obtaining population-level genomic data of museum specimens using ddRAD may still remain feasible when sufficiently large museum collections are available. Prioritising samples based on fragmentation profiles enables the targeting of effort on the most promising samples, enabling production of high-quality data in a cost-efficient manner.

*Main findings and recommendations* :

- ddRAD cannot be routinely applied to large museum collections to obtain population-level genomic data, especially when dealing with heavily-fragmented samples;
- However, despite the challenges of using ddRAD on degraded DNA, we were able to obtain ddRAD seq data from avian samples up to ca. 50 years old, and screening the fragment profiles of the genomic DNA gave good predictions of levels of missing data;
- Such screening is relatively easy to accomplish at minimal cost by any moderately-equipped molecular lab and substantially reduces the risk of both data loss and unnecessary library preparation and sequencing costs;
- The inclusion of data from high-quality fresh samples is important to establish a reference set to aid targeting endogenous sequence data from museum specimens.

*Data availability*: The raw sequencing data from this case study have been deposited at the European Nucleotide Archive (ENA) under project PRJEB59169 with accession IDs ERS14470037 - ERS14470133.

## Case study 6: Single-tube DNA library preparation for ancient bones

### Introduction

Massive parallel sequencing based on sequencing-by-synthesis technologies (Illumina) is an efficient method for generating DNA data from ancient material because it recovers sequences from large amounts of very short DNA fragments. In preparing samples for sequencing, single-tube DNA library protocols circumvent inter-reaction purification steps which require the transfer of DNA solutions to new tubes. They were shown to reduce DNA loss, preparation time and expenses compared to other DNA library preparation methods (Carøe et al. 2018). They also produce comparatively more-complex DNA libraries, i.e. libraries containing a higher proportion of reads mapping uniquely to the reference genome (Carøe et al. 2018). For these reasons, single-tube DNA library preparation methods represent a good option for the shotgun sequencing of ancient museum samples, especially to assess the DNA quality and quantity preserved in series of aDNA specimens. Here, we explore their application to a small series of ancient bones of Bovidae. We applied a single-tube DNA library preparation method that is based on the NEBNext Ultra Kit II DNA Library Prep Kit for Illumina (New England Biolabs) and adapted by Carøe et al. (2018) with ATDC3 adapters to avoid a uracil excision step. Since degraded DNA contains uracil residues resulting from the deamination of cytosines (Briggs et al. 2007), a uracil excision step would further fragment aDNA.

### Materials and Methods

We selected seven bones of Bovidae of different ages (Table 5), from the epipalaeolithic to the late medieval (from 10,200 to 426 years old). Based on morphology, all were suspected to be aurochs (*Bosprimigenius*), but some samples might be large cows (*Bostaurus*). This sampling represents a typical set of challenges for working with natural history collections where specimens are sometimes rare (wild aurochs are extinct), may have different preservation histories and may be misidentified. All manipulations took place in an aDNA lab equipped with UV lamps, under positive air pressure and following best practices recommended for working with ancient DNA (Gilbert et al. 2005, Willerslev and Cooper 2005). UV disinfection was applied before and after each experiment. Clean lab coats, masks, shoe covers and hair caps were worn for each experiment. Gloves were changed after each tube opening. Contacts with other DNA labs were banned (only sterile material was used and access to other labs was not permitted before or during the aDNA analysis). Extraction negatives (samples treated like all others, but without any bone powder inside) were included in all experiments. For tissue sampling, the outer layer of the bone was removed by scraping off its surface using a structured tooth tungsten carbide cutter attached to a hand rotary tool (8100 8v Max Rotary Tool). After 10 min of exposure to UV, 40-75 mg of bone powder was collected by drilling inside the bone fragment using the hand rotary tool at 5000 rpm, with an engraving cutter (1.6 mm). DNA was extracted from the bone powder following the protocol of Dabney et al. (2013b) and was eluted twice in 45 μl of Tris-EDTA buffer with Tween-20. For one specimen (LAST9), four separate extractions were performed. DNA extracts were then evaluated using fluorometry on a Qubit for total double stranded DNA quantification and a Bioanalyzer for fragment-size profiling.

A total of 7 to 51 ng of genomic DNA of each specimen was used as starting material for the ‘Ultra’ single-tube DNA library preparation method described in Carøe et al. (2018). DNA was not sheared. The protocol consists of an end repair step, a ligation of adapters P3 and P5 (final concentration of 0.05 µM each), a fill-in reaction and purification using the MinElute kit (Qiagen). qPCR was used to evaluate DNA quantities available for each specimen for the indexing PCR. Ct values of 11.5 to 15.2 were measured by the qPCR. Based on these Ct values, 10 to 13 cycles were applied to the indexing PCR in order to perform an enrichment that would minimise impacts on the complexity of the DNA libraries (Carøe et al. 2018). The samples were multiplexed with samples from other projects and extraction negative controls in two different libraries of six samples each and sent to Novogene (UK) Company Limited to be sequenced on an Illumina NovaSeq 6000 using a paired-end mode and 150 cycles and to produce 30 Gbp of raw data per library (Suppl. material 6). A total of 12 hours split across three days was necessary for the library preparations.

In total, 225.52 million reads (33.828 Gbp) were generated for the seven specimens and the two controls (Suppl. material 6). Illumina adapters and bad quality bases were removed using AdapterRemoval and options --trimns --trimqualities --minquality 2 (Schubert et al. 2016). Trimmed reads were assembled using PEAR (Zhang et al. 2014), mapped to the nuclear reference genomes of *Bostaurus*, *Homosapiens* and *Musmusculus* (ARS-UCD1.2, GRCh38.p13 and GRCm39, respectively) and to the mitochondrial genome of an auroch (isolate CPC98, GenBank accession number GU985279). Duplicated reads were removed using MarkDuplicates (http://broadinstitute.github.io/picard/). Finally, post-mortem damages were evaluated for data authentication using mapDamage2 (Jónsson et al. 2013) and PMDtools (Skoglund et al. 2014). Two days of analyses were sufficient to perform the bioinformatic analyses with access to a supercomputer.

### Results and Discussion

DNA concentrations after DNA extraction ranged from 0 to 11.8 ng/µl (Table 5). DNA fragment sizes showed a bimodal distribution, with one first peak below 100 bp and a second above 5000 bp. Fragments smaller than 300 bp represented more than 90% of all estimated molarity (Suppl. material 6). These should represent most of the ancient fraction of the DNA extracted. For specimen LAST9, 0.5 ng/µl DNA was detected in one extract, while no DNA was detected in the three other extracts. Two days of five hours were necessary for all DNA extractions and the price per sample was estimated at ca. €35 (including taxes, but excluding manpower). The costs of the library preparation and the sequencing were estimated at €55 and €75 per sample, respectively (including taxes, but excluding manpower). Proportions of sequenced reads mapped to the bovine genome provide an indication of the proportion of endogenous DNA and varied from 0.02% (specimen from the Roman times) to 7.84% (late medieval specimen). Compared to the reads mapped to the human or to the mouse genomes, those mapped to the bovine genome represented a higher percentage of all reads in all samples but one (LAST4). They showed a narrower size distribution, concentrated below 100 bp and shorter insert sizes (Table 5). They also showed patterns of DNA degradation in mapDamage2 (Jónsson et al. 2013) and higher post-mortem damage scores (Suppl. material 6) in PMDtools (Skoglund et al. 2014). These features are indicative of ancient endogenous DNA and were not observed in the negative controls (Table 5). Between 1.8 and 96% of the bovine mitochondrial genome was recovered, depending on the samples, with some regions covered from 0 to 32 times.

**Data authentication**

The single-tube library preparation protocol applied here (Carøe et al. 2018) provided DNA reads for all seven specimens tested, with varying proportions of DNA that mapped to the reference genome of *Bostaurus*. The authentication of these reads is critical for downstream analysis and should test both the ancient and endogenous origin of the reads filtered for analysis. This includes checking signatures of degradation including nucleotide alterations and DNA size profiles (Hofreiter et al. 2001, Renaud et al. 2019) and comparisons with reads from negative controls and reads mapped to other genomes. Here, most reads mapped to the bovine genome were smaller than 100 bp and in the same range as those obtained by Carøe et al. (2018) from eight historic grey wolf skins of 90 to 146 years old (40-180 bp with an average of around 60 bp). Estimating insert sizes when mapping paired reads is useful to evaluate the size distribution of DNA fragments in the library. Thus, even though it is more expensive, generating paired-end reads and reads larger than the average short read lengths revealed by the Bioanalyzer (i.e. 100 cycles or more), enables the exclusion of reads obtained from longer DNA fragments, which may correspond to recent contaminations. It is also important to filter out contaminant reads that still map to the target genome. Indeed, short contaminant fragments can map to evolutionary more conserved regions of divergent genomes. Thus, removing reads that map both to the target genome and other divergent genomes is a useful precaution. Competitive mapping can address this by mapping raw sequencing data to a concatenated reference composed of the target species' genome and other possible contaminant genomes, such as the human genome. The sequences aligned only to the target part of the concatenated reference genome can be kept for downstream analyses (Feuerborn et al. 2020). Further authentication would include a completely independent analysis (from extraction to sequencing) to check the congruence of the results (Andreeva et al. 2022).

*Main findings and recommendations*:

- Streamlined single-tube DNA library preparation methods adapted for degraded DNA and followed by shallow shotgun sequencing are useful for estimating the percentages of ancient endogenous DNA recoverable from DNA extracts;
- Data authentication should integrate as many aspects of the endogenous DNA as possible. Comparisons with controls (extraction negatives and unrelated genomic data) are crucial to assess the risk of including contaminant DNA in the analysis and to guide steps to filter them out;
- DNA fragment-size profiles of the DNA extracts are indicative of the presence of degraded DNA, but sequencing is necessary to evaluate percentages of endogenous DNA.

*Data availability*: The raw sequencing data for this case study have been deposited at the European Nucleotide Archive (ENA) under project PRJEB59185 with accession IDs ERS14471070 - ERS14471078.

## Concluding remarks

The continually evolving landscape of sequencing platforms and chemistries is resulting in an ever-expanding set of opportunities for unlocking the genomic resources held in natural history collections and there is a general increase in the feasibility of museum specimen sequencing. With the rapid expansion in the field of museomics, comes a pressing need for the ongoing development, sharing and adoption of best practices. Areas of particular importance include establishment of appropriate facilities, workflows and data verification steps to minimise risks of contamination and sampling guidance which supports optimal utilisation of museum specimens for genomic research. Another area of general importance is attention to ethical issues associated with the use of specimens for genomic science; many collections pre-date contemporary permit conditions or restrictions. Guidelines for ethical issues associated with sampling specimens for genomic analysis are mostly developed for human tissues and archaeofaunal remains (Prendergast and Sawchuk 2018, Pálsdóttir et al. 2019); further dialogue (e.g. Canales et al. (2022)) and policy development regarding best practice for genomic sampling of wider natural history collections are needed.

## Supplementary Material

5840DFD2-4B2E-5184-8351-D949BD548125 10.3897/BDJ.11.e102317.suppl1 Supplementary material 1 hDNA laboratory equipment list Data type equipment list Brief description List of equipment, its purpose and exemplar manufacturer, model and cost (as of 2022) for establishing an hDNA facility. File: oo_802633.xlsx https://binary.pensoft.net/file/802633 Giada Ferrari

F965914F-7683-5884-9E89-2C8C71AB8346 10.3897/BDJ.11.e102317.suppl2 Supplementary material 2 Case study 2 metadata Data type Accession information and DNA/sequence quality and quantity statistics Brief description Spreadsheet containing sample ID, accession details, collecting data, preservation method, laboratory protocols used and DNA quantity recovery and sequence quality statistics. File: oo_861124.xlsx https://binary.pensoft.net/file/861124 Lore Esselens

D6B12AF0-805E-5855-9D6B-7613164E389E 10.3897/BDJ.11.e102317.suppl3 Supplementary material 3 Case study 3 accession details Data type Spreadsheet of accession details Brief description Accession details and sample type of the specimens analysed in Case study 3. File: oo_867176.xlsx https://binary.pensoft.net/file/867176 Steven Janssens

8FA40478-0BD7-5BD1-A925-6D891229C8AE 10.3897/BDJ.11.e102317.suppl4 Supplementary material 4 Case study 4 accession details Data type Spreadsheet of accession details Brief description Accession details, links to living and herbarium specimens databases, DNA concentration and library preparation methods. File: oo_803260.xlsx https://binary.pensoft.net/file/803260 Giada Ferrari

E29F4E83-A9D6-5987-ABE7-E27C351418B3 10.3897/BDJ.11.e102317.suppl5 Supplementary material 5 Case study 4 mapping statistics Data type Spreadsheet with mapping statistics for hybridisation capture Brief description Read statistics (raw reads, reads mapping to target loci, read clonality, coverage). File: oo_871797.xlsx https://binary.pensoft.net/file/871797 Giada Ferrari

0817F41A-FA48-50C3-8AC4-1EA42024ABB1 10.3897/BDJ.11.e102317.suppl6 Supplementary material 6 Case study 6 sample, DNA and read data description Data type Table with descriptive data Brief description Sample information, DNA extracts evaluation and DNA reads description. File: oo_917809.docx https://binary.pensoft.net/file/917809 Gontran Sonet

## Figures and Tables

**Table 1.** Selected papers outlining recent progress, breakthroughs and protocol developments that support the more routine recovery of genomic data from museum specimens

**Study taxon**	**Tissue type**	**Specimen ages (yrs)**	**Approach(es)**	**Key finding**	**Reference**
Plants	Dried herbarium specimens	2-182	Multi-locus nuclear sequence capture	Large-scale study of 7608 specimens using angiosperm 353 target capture baits; DNA yield poor predictor of sequencing success, plant family strongest predictor of success, successful recovery of old specimens from tropical climates	Kates et al. (2021)
Plants	Dried herbarium specimens	NA	NA	Protocols and best practices for working with ancient and historical plant DNA. Includes laboratory set-up, DNA isolation, sequencing library preparation and bioinformatic analyses	Latorre et al. (2020)
Plants	Dried herbarium specimens	Up to 280	Shotgun sequencing	DNA in herbarium specimens degrades faster than in ancient bone. Both fragmentation and deamination accumulate over time	Weiß et al. (2016)
Plants	Dried herbarium specimens	most < 20 yrs, 165 samples > 50 yrs; oldest 153 yrs	Shotgun sequencing	Large-scale genome skimming study of 2051 herbarium specimens recovering plastome and rDNA sequences including standard plant barcodes	Alsos et al. (2020)
Fungi	Rust fungi on dried herbarium specimens	Up to 187	Amplicon-based rDNA sequencing	Protocol development and application to track dynamics of plant pathogens through time sampled from herbarium specimens	Bradshaw et al. (2023)
Fungi	Fungarium specimens	Less than 20	Whole genome sequencing	Generation of draft genome assemblies possible and of value for enhancing resolution of fungal phylogeny	Dentinger et al. (2016)
Birds	Avian skins	up to ca. 150	Whole genome sequencing	Step-by-step guide to workflow and protocols, including steps taken to minimise risks of contamination	Irestedt et al. (2022)
Mammals (mephitids, rodents, marsupials)	Dried museum skins	50-120	Shotgun sequencing	Comparison of DNA yields and bacterial contamination levels in commonly-sampled museum mammalian tissues (bone, claw, skin and soft tissue) and implications for sampling strategies	McDonough et al. (2018)
Mammals (grey wolf)	Dried museum skins	90 - 146	Shotgun sequencing	“Single-tube” DNA library preparation methods including adaptations for degraded DNA increase library complexity, yield more reads that map uniquely to the reference genome and reduce processing time compared to other Illumina library preparation methods	Carøe et al. (2018)
Mammals (bison and horse)	Bone	Up to 40,680	Shotgun sequencing	A more accessible single-stranded genomic library preparation method optimised for aDNA	Kapp et al. (2021)
Mammals (dogs and mammoths)	Bone	Up to 37,080	Shotgun sequencing	Competitive mapping of raw sequencing data to a concatenated reference composed of the target species' genome and the genome of possible contaminants contributes to filtering out contamination from ancient faunal DNA datasets with limited losses of true ancient data	Feuerborn et al. (2020)
Mammals (Cricetidae, rodents, deer mouse)	Frozen liver tissue	17 to 41	Whole genome sequencing	Linked-read or “synthetic long-read” sequencing technologies provide a cost-effective alternative solution to assemble higher quality de novo genomes from degraded tissue samples	Colella et al. (2020)
Insects (Phylinae; plant bugs)	Abdomen	1 to 54	DNA bait capture	Inexpensive data generation to produce sufficient amount of data to assemble the nuclear ribosomal rRNA genes and mitochondrial genomes	Knyshov et al. (2019)
Insects (Apidae, bumble bees)	Leg	18 to 131	Shotgun sequencing	DNA in entomological specimens in NHC highly degraded, process age dependent with a roughly linear reduction in fragment length over time after strong initial fragmentation	Mullin et al. (2023)
Insects (Culicidae, mosquitoes)	Whole specimens	33 to 84	Shotgun sequencing	Minimally damaging extraction method for building libraries for Illumina shotgun sequencing	Korlević et al. (2021)
Insects (Lepidoptera)	Leg	Average 50 years	Sequel CO1 barcoding	Demonstration of utility of PacBio Sequel platform for recovery of full length CO1 barcodes	D'Ercole et al. (2021)

**Table 2.** An overview of the DNA extraction kits tested on fruit flies and hoverflies.

**QIAGEN kit (50 samples)**	**Spin column**	**Range of DNA fragment sizes (according to manufacturer’s instructions)**	**Expected DNA yield (according to manufacturer’s instructions)**
DNeasy Blood and Tissue Kit	DNeasy spin column	100 bp-50 kb	6-30 µg
QIAamp Micro Kit	QIAamp MinElute column	< 30 kb	< 3 µg
QIAamp Mini Kit	QIAamp Mini spin column	< 50 kb	4-30 µg
DNeasy Blood and Tissue Kit	MinElute column (MinElute PCR Purification Kit)	70 bp-4 kb	< 5 µg

**Table 3.** Number of processed collection vouchers from three Tephritidae and two Syrphidae genera.

**Collection**	**Genus**	**Number of specimens**
Tephritidae	*Bactrocera*	16
Tephritidae	*Dacus*	197
Tephritidae	*Ceratitis*	411
Syrphidae	*Eristalinus*	83
Syrphidae	*Melanostoma*	25

**Table 4.** Details of *Rhododendron* herbarium samples used in this study including collection and accession numbers, as well as library protocols used. RHD002 and RHD007 herbarium specimens relate to the same single individual in the living collection, as do RHD016 and RHD018, respectively. Two samples (RHD011 and RHD018) had sequencing libraries prepared using two different protocols. Fresh samples from the living collection were also collected for all individuals. DNA fragment size distribution: size as stated, except bimodal which means one peak of < 1000 bp and one peak of approximately 1-20 kbp. ssDNA = single-stranded DNA library, NEB = NEBNext Ultra II library with sonicated DNA. *All sequencing libraries for the *living collection* were prepared using NEBNext Ultra II kits with sonicated DNA.

**Sample**	**Species**	**Subspecies**	**RBGE herbarium collection number**	**Specimen date**	**RBGE living collection accession number**	**DNA fragment size distribution**	Library protocol(s) for herbarium samples*
RHD002	*Rhododendronjavanicum*	*kinabaluense*	E00421003	2010	19801291A	bimodal	NEB
RHD003	*R.javanicum*	moultonii	E00294943	2009	20110223A	bimodal	NEB
RHD005	*R.javanicum*		E00328126	2009	19672627A	100-1000 bp	NEB
RHD006	*R.javanicum*	*brookeanum*	E00328133	2009	19801298C	bimodal	NEB
RHD007	*R.javanicum*	*kinabaluense*	E00328548	2009	19801291A	bimodal	NEB
RHD008	*R.javanicum*	*palawanense*	E00294512	2008	19922762B	bimodal	NEB
RHD009	*R.javanicum*	*cladotrichum*	E00294755	2007	19913084A	bimodal	NEB
RHD011	*R.javanicum*	*palawanense*	E00954297	1998	19922772	bimodal	ssDNA, NEB
RHD013	*R.javanicum*	*javanicum*	E00954260	1990	19730741	< 500 bp	ssDNA
RHD016	*R.javanicum*	*javanicum*	E01016321	1982	19680840	< 500 bp	ssDNA
RHD017	*R.javanicum*	*kinabaluense*	E01016323	1981	19690955	< 500 bp	ssDNA
RHD018	*R.javanicum*	*javanicum*	E01016322	1972	19680840	< 500 bp (+tail)	ssDNA, NEB

**Table 5.** Bovid bone sample information, isolated DNA concentrations and proportions of reads mapped to target and possible contaminant full genomes. ID: Tissue sample identification; conc: DNA concentration in the DNA extract measured using Qubit; mapped: percentages of the deduplicated paired-end reads mapping to the reference genomes of *Bostaurus*, *Homosapiens* and *Musmusculus* (separated by “/”); short: percentages of mapped reads smaller than 100 bp; long: percentages of mapped reads longer than 300 bp (with insert between the paired reads); Neg1 and Neg2: negative DNA extracts processed for each library.

**ID**	**Epoch**	**Conc**	**Mapped**	**Short**	**Long**
		**ng/µl**	% **N raw**	% **< 100 bp**	% **> 300 bp**
**LAST1**	Roman period	1.4	0.157/0.06/0.077	83.43/2.93/1.79	0.52/10.05/10.58
**LAST2**	Roman period	11.8	0.644/0.01/0.004	92.87/36.08/39.93	0.31/17.67/7.72
**LAST3**	Roman period	2.6	1.674/0.055/0.073	85.84/14.15/5.65	0.42/6.14/9.98
**LAST4**	Roman period	3.7	0.02/0.03/0.054	72.39/3.7/1.88	1.38/7.71/10.1
**LAST5**	Roman period	5.2	0.123/0.017/0.005	88.27/11.29/19.94	0.39/26.95/9.76
**LAST7**	epipaleolithic	7.8	0.033/0.01/0.003	98.01/13.29/21.84	0.49/24.7/10.18
**LAST9**	late medieval	0.5	7.844/0.179/0.169	84.91/16.83/6.91	0.33/8.08/11.46
**Neg1**	NA	0	0.058/1.55/0.417	59.44/3.18/0.31	3.89/29.07/13.07
**Neg3**	NA	0	0.278/2.828/2.382	18.01/1.53/0.92	5.64/2.47/12.43
