# Supplementary material for: Developing the Protocol Infrastructure for DNA Sequencing Natural History Collections
Source: Biodivers Data J. 2023 Oct 27;11:e102317. doi: 10.3897/BDJ.11.e102317 (PMC10848826; doi:10.3897/BDJ.11.e102317)
Supplement: Supplementary material 6 — Case study 6 sample, DNA and read data description [file bdj-11-e102317-s006.docx]

Case study 6 sample, DNA and read data description

| **ID** | **geo origin** | **bone** | **lib** | **qty** | **% < 300 bp** | **N reads** | **degradation** |
| --- | --- | --- | --- | --- | --- | --- | --- |
|  |  |  |  | **ng** | **molarity** | **raw pairs** | **PMD score** |
| LAST1 | Tongeren | humerus | 3 | 19 | 99.735 | 21627111 | 1.14/-1.03/-1.24 |
| LAST2 | Tongeren | femur | 1 | 47 | 99.945 | 39539991 | 0.58/-0.65/-0.76 |
| LAST3 | Oudenburg | femur | 3 | 37 | 99.818 | 19129639 | 1.04/-0.9/-1.19 |
| LAST4 | Oudenburg | radius | 3 | 51 | 99.766 | 22583455 | 0.32/-1.03/-1.25 |
| LAST5 | Oudenburg | radius | 1 | 47 | 99.857 | 31144622 | 0.92/-0.95/-1.03 |
| LAST7 | Matignolle | phalanx 1 | 1 | 47 | 99.767 | 64854647 | 0.88/-0.97/-1.02 |
| LAST9 | Brussels | horn core | 3 | 7 | 90.658 | 26645199 | 0.32/-0.86/-1.15 |
| Neg1 | NA | NA | 1 | 0 | 0.000 | 310028 | 0.37/-0.99/-1.16 |
| Neg3 | NA | NA | 3 | 0 | 0.000 | 848988 | -0.75/-1.08/-1.24 |

ID: Tissue sample identification; geo origin: geographic origin; lib: DNA library identification; qty: DNA quantity used for library preparation; % <300 bp: percentage of DNA molecules smaller than 300 bp in the DNA extract based on the Bioanalyzer DNA high sensitivity fragment analysis; N reads: number of Illumina paired-end reads in the raw data; PMD: post mortem damage (of reads mapped to the reference genomes of *Bos taurus* / *Homo sapiens* / *Mus*

*musculus*); Neg1 and Neg2: negative DNA extracts processed together with library 1 and 2, respectively.
